# What determines FinTech success?—A taxonomy-based analysis of FinTech success factors

**DOI:** 10.1007/s12525-023-00626-7

**Published:** 2023-05-19

**Authors:** Oliver Werth, Davinia Rodríguez Cardona, Albert Torno, Michael H. Breitner, Jan Muntermann

**Affiliations:** 1 OFFIS - Institute for Information Technology, Escherweg 2, 26121 Oldenburg, Germany; 2 Information Systems Institute, Leibniz University Hannover, Königsworther Platz 1, 30167 Hanover, Germany; 3 Faculty of Business and Economics, Chair of Electronic Finance and Digital Markets, Georg-August-Universität Göttingen, Platz Der Göttinger Sieben 5, Göttingen, 37073 Germany; 4 Faculty of Business and Economics, Chair of Financial Data Analytics, Augsburg University, Universitätsstrasse 16, 86159 Augsburg, Germany

**Keywords:** FinTech success factors, Taxonomy-based analysis, FinTech business models, G29, O33, O39

## Abstract

Value creation in the financial services sector has been fundamentally transformed by digitally born financial technology (FinTech) companies. FinTech companies synthesize information systems with financial services. Given its disruptive power, the FinTech phenomenon has received great attention in academic research, practice, and media. Still, limited systematic research provides a structure and holistic view of FinTechs’ success. Aiming to enhance understanding of the factors enabling FinTech success, we classify success factors across extant scientific literature on distinct FinTech business model archetypes. Our analysis reveals that the “cost–benefit dynamic of the innovation,” “technology adoption,” “security, privacy, and transparency,” “user trust,” “user-perceived quality,” and “industry rivalry” are crucial factors for FinTech success and can be seen as “grand challenges” for the FinTech ecosystem. In addition, we validate and discuss our findings with real-world examples from the FinTech industry and two interviews with stakeholders from the FinTech ecosystem. Our study contributes to the knowledge of FinTechs by providing a classification system of success factors for practitioners and researchers.

**Supplementary Information:**

The online version contains supplementary material available at 10.1007/s12525-023-00626-7.

## Introduction

Success and its associated concepts have been identified for their high importance in information systems (IS) and management research (e.g., Petter et al., 2013; Thompson et al., 2018). Success can be determined through a collection of relevant factors that a company or industry should focus on to achieve a competitive performance (Petter et al., 2013; Rockart, 1979). Given the interconnection between success and competitive performance, recent studies have found empirical evidence supporting the association between venture success and “the design or architecture of the value creation, delivery, and capture mechanisms” (Teece, 2010, p. 172) of a company, framed through the conceptualization of its underlying business model (e.g., Böhm et al., 2017; Weking et al., 2019).

In the financial industry, the value creation process has been fundamentally transformed into innovative business models that integrate digital technologies and concepts (Imerman & Fabozzi, 2020). Furthermore, this convergence between IS and financial services is enhanced through “born-digital” financial technology companies (FinTechs). Due to their digital nature, FinTechs can create new value propositions, e.g., increasing financial inclusion and decreasing income inequality (Demir et al., 2020; Lagna & Ravishankar, 2021) or reshaping the financial system and the monetary policy implementation through financial disintermediation (Mumtaz & Smith, 2020). Given this social and economic disruptive power, the sustainability of the FinTech phenomenon is receiving increasing attention in academic research and growing relevance in practice (Klein et al., 2020; Omarova, 2019).

Looking closely at the FinTech market, the number of FinTechs rose from around 12,000 in 2018 to 26,000 in November 2021 (Statista, 2021a). In addition, FinTech adoption rates have significantly increased in several countries like the USA and the UK (Insider Intelligence, 2021; Statista, 2021a). Pre-COVID-19 pandemic investments in these ventures raised significantly until 2019 to 215.1 billion US Dollars (Statista, 2021b). Most recent data conclude that the investment volumes are higher than the pre-pandemic level (226.5 billion US Dollars in 2021) and seem to continue in 2022 (107.8 billion US Dollars after the first half of 2022) (Statista, 2021b). Therefore, the FinTech market is still an attractive business field for investors and founders, and customers.

However, failure rates of FinTechs are approximately 75% (The Fintech Mag, 2022) and 87%, for example, in Germany within 3 to 6 years (Stuckenborg & Leker, 2019) after the founding. In the past, the role of FinTech and the survival of such companies were described as a “FinTech Bubble” that would burst (Dratva, 2020, p. 66). Many FinTech companies face market or product risks (Buckley & Webster, 2016) and a loss of valuation, like in the case of Klarna (Fintech Magazine, 2022). These situations challenge FinTechs to maintain their survival in the market. Accordingly, (new) FinTechs must meet success factors (SFs) to remain attractive to investors and customers, especially in the first years of founding. FinTech survival through a provision of a constant flow of venture capital, high liquidity, and profits can avoid failure in this innovative service ecosystem (Stuckenborg & Leker, 2019).

SFs for FinTechs have been previously stated as an interesting research field that contributes to developing successful FinTech business models (Gomber et al., 2017). As a result, well-grounded knowledge and the consideration and possible prioritization by managers and investors of SFs result in a constant flow of venture capital and retain the survival of FinTech business models (e.g., Kolokas et al., 2022; Laidroo & Avarmaa, 2020; Nicoletti, 2017). In contrast, the still incipient integrative academic literature on success research in FinTech indicates a fragmented understanding of the SFs that contributes to successful FinTech business models (e.g., Imerman & Fabozzi, 2020; Werth et al., 2019). Therefore, these businesses and entrepreneurial SF need to be discovered comprehensively over the heterogeneous FinTech market. An examination and well-justified identification of potentially relevant SF for FinTechs is required. Guided by these motivations, the following research question (RQ) is addressed in this study:

- RQ: Which theoretically grounded factors are potentially relevant for FinTech venture success across distinct FinTech archetypes and business model dimensions?

To answer our RQ, we create a literature search-based taxonomy according to the methodology proposed by Nickerson et al. (2013). This allows us to detect theoretically grounded factors previously identified by past research as relevant for FinTech venture success. We contribute to the FinTech and IS literature by providing a taxonomy that can explain the differences and similarities of objects and uncover and classify non-existent object configurations or knowledge gaps (Muntermann et al., 2015). As a result, the taxonomy can serve as an artifact that can be used to solve practical problems, i.e., the identification of potentially relevant SFs for FinTechs (Kundisch et al., 2021). We utilize the scientific literature about SFs for FinTech venture success and classify it across distinct FinTech business models identified by Eickhoff et al. (2017). Following the guidelines for taxonomy evaluation by Kundisch et al. (2021), we demonstrate the applicability of our taxonomy with a case-based taxonomy validation and discussed our findings for usefulness with two individuals from the FinTech ecosystem. From a theoretical perspective, our results and conclusions can support further theory-building processes for conceptualizing SFs across FinTech business models and serve as a vantage point for further systematic investigations in this area. Practitioners can use our results to compare their business models and consider realigning as well as prioritizing the allocation of resources in key business areas. In addition, our study serves as a discussion and supplementary information base among stakeholders, e.g., venture capitalists, about relevant SFs. In summary, our study generates a meaningful knowledge base for all involved and interested stakeholders in the FinTech ecosystem.

The rest of this paper is structured as follows: Firstly, we define SFs and describe their relationship to FinTech business models. Afterward, we explain our research methodology and data collection procedure. Next, real-world examples from the FinTech industry are discussed using archetypes and dimensions of the FinTech business. To confirm the relevance of the SFs provided, we present and discuss our findings with two individuals from the FinTech ecosystem. Furthermore, theoretical, methodological, and practical implications are presented. Lastly, we provide limitations, future research directions, and concluding remarks.

## Domain background about success factors and FinTechs

Success is defined as the “accomplishment of an aim or purpose” (Oxford Dictionaries, 2022) or the achievement of “a satisfactory/favorable outcome” (Ain et al., 2019, p. 7). SFs, in the business context, are certain issues that determine a firm’s success with its products or services in the market. We follow Thompson et al., (2018, p. 75), who define SFs as “the strategy elements, product and service attributes, operational approaches, resources, and competitive capabilities essential to surviving and thriving in the industry.” Organizations directly influence these factors, which corresponds to an internal view of the SFs. In line with other definitions, including those of Rockart (1979) and David and David (2017), we also consider external factors, e.g., environmental factors, as relevant to success because not considering these factors could lead to detrimental effects on the competitive success of FinTechs. While SFs are often labeled in literature with the prefix “critical” (e.g., Freund, 1988; Rockart, 1979) or “key” (e.g., David & David, 2017; Thompson et al., 2018), we use the term “success factor(s)” without any prefix, because we do not intend to rank these factors. Instead, our goal is to provide a well-justified list of relevant factors that influence the success of FinTechs.

SFs should be few and controllable because they aim to measure a firm’s competitive advantage or success within an industry, e.g., through strategic analysis tools (Thompson et al., 2018). However, the main challenge in developing these success measuring tools, e.g., the Competitive Profile Matrix (CPM), is to identify the respective underlying SFs of the industry (Bhattacharjee, 2015). For example, research on SFs for technology-based startups has identified some common categories of SFs, e.g., “organizational, individual and external factors” (e.g., Santisteban & Mauricio, 2017), “advantage of the radical innovation, characteristics of the organization, characteristics of the entrepreneur” (e.g., Groenewegen & de Langen, 2012), or “accounting, market, and stakeholder-based factors” (e.g., Soto-Simeone et al. 2020). While these abstract factors may influence tech startups in general, there is a consensus that industry-specific research is required to determine the nature of SFs in each specific business context (Gazel & Schwienbacher, 2020; Soto-Simeone et al. 2020). Hence, this study addresses the challenge of identifying relevant SFs in the FinTech industry across different business model archetypes.

The phenomenon known as FinTech can be described as financial innovations enabled by information technology (IT) resulting in new financial instruments, services, and/or intermediaries (Arner et al., 2016). To characterize the different levels of novelty in FinTech, Gomber et al. (2017) describe two degrees of FinTech innovation, i.e., “sustaining FinTech” and “disruptive FinTech.” Sustaining FinTech comprises financial services incumbents incorporating information technologies to maintain their position in the market, while disruptive FinTech comprises new market competitors providing novel digital financial products and services. In addition, Gomber et al. (2018) explained that besides sustaining and disruptive FinTech, incremental, product-supplementing, and radical, new product-generating, FinTech innovations exist. We decided to follow the definition of FinTech by Eickhoff et al., (2017, p. 2), given that this definition is conceptualized at the business model level and is thereby consistent with the level and context of our analysis. Consequently, we understand FinTech as the companies that “operate at the intersection of financial products and services and IT. They are usually relatively new companies (often startups) with their innovative product or service offerings” (Eickhoff et al., 2017, p. 2). In parallel, research on SFs for technology-based startups has identified some common categories of SFs compared to traditional startups, e.g., “organizational, individual, and external” factors (e.g., Santisteban & Mauricio, 2017) or “advantage of the radical innovation, characteristics of the organization, and characteristics of the entrepreneur” (e.g., Groenewegen & de Langen, 2012). While these abstract factors may influence tech startups, there is a consensus that industry-specific research is required to determine the nature of SFs in each specific business context, e.g., within financial services (Gazel & Schwienbacher, 2020; Soto-Simeone et al. 2020). Accordingly, FinTech startups differ from traditional business models and high-tech startups because of their integrative nature in technological, entrepreneurial, and finance business contexts. For example, past research indicates that incumbent partnerships with traditional financial services providers are relevant since they are important for the short- and middle-term survival to acquire a sustainable pool of customers (Laidroo & Avarmaa, 2020; Werth et al., 2019).

Depending on the value configuration of the underlying FinTech business models, FinTech innovation can take the form of (i) new customizable, easy-to-use, and efficient products; (ii) new forms of production that minimize risks or costs; (iii) new customer interfaces or distribution channels; (iv) new markets and customer segments; and/or (v) new technology-driven business models (Eickhoff et al., 2017; Gomber et al., 2017). To better understand the value creation mechanisms of FinTechs, academics have developed diverse taxonomies about FinTech business models from different perspectives. Gimpel et al., (2018) presented a taxonomy of the characteristics of consumer-oriented FinTech business models using statistical techniques. A study from Beinke et al. (2018) developed a taxonomy to identify the business model elements in FinTechs that use Blockchain technology. Nagel et al. (2019) proposed a taxonomy of these Blockchain-based business models specifically for smart cities. Conversely, Drasch et al. (2018) identified 13 dimensions of cooperation patterns between FinTechs and banks using a taxonomic approach, and Imerman and Fabozzi (2020) presented a taxonomic structure of the Fintech ecosystem by unifying the concept of digital transformation with the relevant functional areas and emerging technologies for FinTech. Meanwhile, using a clustering-based approach, Eickhoff et al. (2017) empirically examined 2040 FinTechs from the Crunchbase database. As a result, Eickhoff et al. (2017) identified ten clusters of FinTech business model archetypes, e.g., cryptocurrency or payment services, that serve as a base for our analysis of FinTech SFs. These FinTech business model archetypes are a starting point for our taxonomy development process.

## Methodology and data collection procedure

**Fig. 1:**
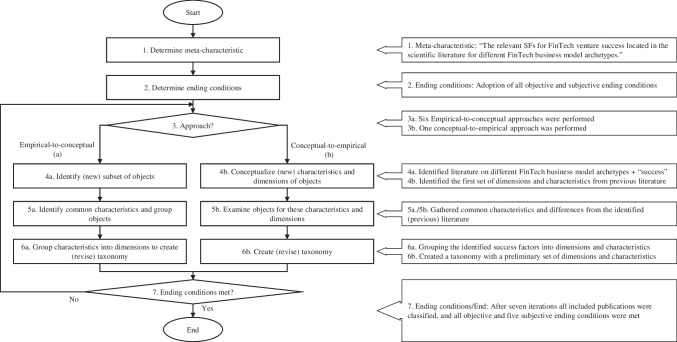
Adapted taxonomy development method by Nickerson et al., (2013)

We conduct a taxonomy-based content analysis to generate insights into the factors enabling FinTech success. Figure 1 thereby shows the adapted taxonomy development method of Nickerson et al. (2013) and the methods used, which serves as the foundation of our research approach. We follow the methodological approach of Nickerson et al. (2013) because this taxonomic approach is connected to an iterative nature aimed at reducing the complexity of scattered information through the integration of theoretical knowledge (deductive iteration) and empirical evidence (inductive iteration). Therefore, it is a suitable approach to guide our literature-based study to distillate findings and structure the existing knowledge on FinTech success (Szopinski et al., 2019). Generally, in empirical-to-conceptual iterations, “the researcher identifies a subset of objects that he/she wishes to classify” (Nickerson et al., 2013, p. 345). Conceptual-to-empirical approaches aim to conceptualize the dimensions of a (preliminary) taxonomy from previous knowledge, e.g., literature, without examining actual objects (Nickerson et al., 2013).

**Fig. 2:**
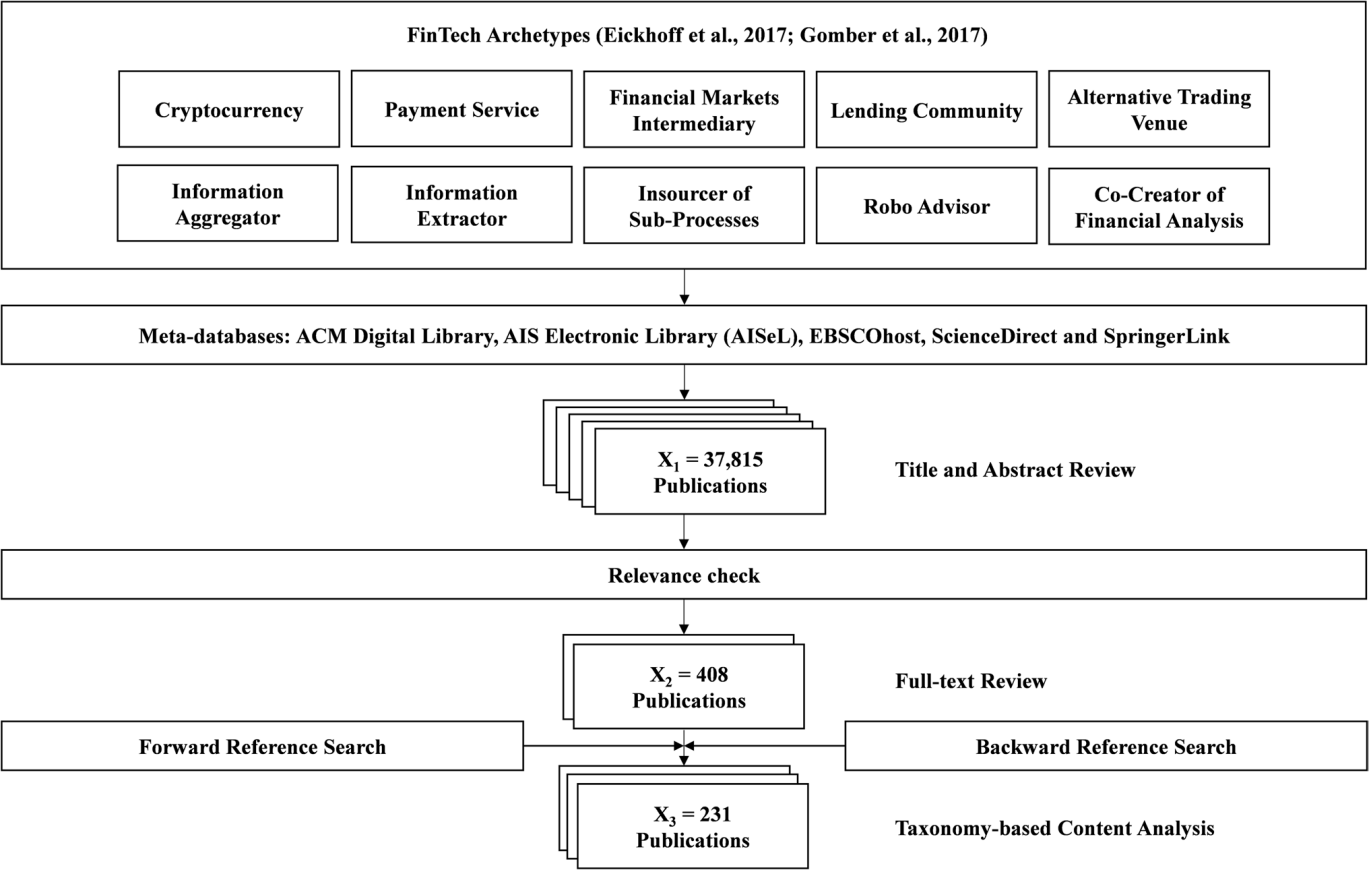
Literature review procedure

First, the meta-characteristic needs to be determined, which is “the most-comprehensive characteristic” and “serves as base for all other characteristics” (Nickerson et al., 2013, p. 343). Furthermore, the intended users, i.e., researchers and practitioners interested in FinTech business models and SFs, must be considered. To be as comprehensive as possible regarding the FinTech market, we use the FinTech business model archetypes presented by Eickhoff et al. (2017). Guided by these facts and our RQ, we define our meta characteristic as “the relevant SFs for FinTech venture success located in the scientific literature for different FinTech business model archetypes.” After outlining the meta-characteristic, ending conditions must be determined, ending the taxonomy development if met. We adopted all objective (e.g., “no dimensions or characteristics were merged or split in the last iteration of the taxonomy process”) and all subjective (e.g., extendable nature of the taxonomy) ending conditions according to Nickerson et al. (2013). We followed a conceptual-to-empirical approach as a starting point and subsequently refined the taxonomy by examining and classifying a set of representative objects obtained through ten lateral systematic literature reviews corresponding to the ten FinTech business model archetypes developed by Eickhoff et al. (2017), as shown in Fig. 2. Based on the guiding principles of Webster and Watson (2002) and Wohlin (2014), we performed both a keyword and a forward–backward literature search (Hinde & Spackman, 2015), which we further complemented with an author and similarity search as shown below:

The determination of the representative keywords has been accomplished by considering the FinTech business model archetypes established by Eickhoff et al. (2017) and the keywords identified by Cziesla (2015) throughout their literature analysis of the digital finance sector. We also used these keywords as umbrella terms to identify additional keywords mentioned in the academic literature. We searched for scientific articles using the set of keywords in Table 6 in the appendix in conjunction with the search term “success” in different databases.

Given the interdisciplinary nature of the topic, our examination of scientific literature covered a total of 118 peer-reviewed scientific journals and ten conference proceedings in the fields of IS, finance, and banking (BA-FI), strategic management (SM), as well as technology, innovation, and entrepreneurship (TIE). The scientific journals and conference proceedings were retrieved through five publisher databases (Sturm & Sunyaev, 2017): ACM Digital Library, AIS Electronic Library (AISeL), EBSCOhost, ScienceDirect, and SpringerLink. In addition to defining the relevant keywords, we delineated a set of inclusion criteria to be fulfilled. To be included in our taxonomy-based content analysis, scientific articles must (i) allude to determinant factors of success for any FinTech business model archetype (Eickhoff et al., 2017) and fulfill the following inclusion criteria: (ii) be published in English, (iii) between January 2008 and June 2020, and (iv) in recognized peer-reviewed academic business (i.e., BA-FI, SM, TIE) and IS journals or conference proceedings (Hennig-Thurau et al., 2022).

The selection process to identify the representative sample of scientific articles to be examined in the content analysis followed the same systematic procedure depicted previously in Fig. 2. In the stage corresponding to the keyword-based search, we observed scientific articles containing our keywords in their abstracts and/or title. In this initial set (*X*_1_), we identified 37,815 potentially eligible research articles fulfilling the first three inclusion criteria. In the second stage, the eligibility of the initial set was narrowed down by eliminating duplicate articles and applying a relevance check. This approach allowed us to limit our initial sample to a set (*X*_2_) of 408 scientific articles, potentially fulfilling the fourth inclusion criterion. To ensure that only articles relevant to the success of FinTech are included in the final set of articles, we performed a full-text review of the potentially relevant articles. After excluding all irrelevant articles for our analysis (e.g., non-FinTech relevant articles, randomly mentioned in the abstract, keywords, or articles focusing only on technical aspects of digital technologies), our sample was reduced to 215 relevant articles.

To enhance this pool of relevant articles, we conducted forward, backward, author, and similarity searches. The forward, author, and similarity search strategies used Google Scholar. With the forward search, we identified five additional relevant articles that quoted the scientific articles found in the former search stage (Wohlin, 2014). With the author search, we found five additional relevant articles by the most important authors. With the similarity search, we found three additional relevant articles similar to the most important articles found before. The backward search was applied by analyzing the reference lists of the articles obtained from the previous search phases; through this approach, we identified four additional relevant articles. Within these procedures, we found a total of 17 additional articles, which fulfill all inclusion criteria.

As a result, we identified a final sample (*X*_3_) of 231 research articles fulfilling all inclusion criteria (alternative trading venues: *n* = 87, payment services: *n* = 76, insourcer of sub-processes: *n* = 20, lending community: *n* = 19, cryptocurrency: *n* = 15, co-creator of financial analysis: *n* = 7, robo-advisor: *n* = 4, and information aggregator: *n* = 3). For the FinTech business model archetypes “financial markets intermediary” and “information extractor,” we did not find articles that fulfill all of our inclusion criteria. A list of all relevant papers identified and analyzed in our study is available in the appendix, Table 5.

## Taxonomy-based content analysis of FinTech success factors

Our taxonomy-based analysis aims at structuring the knowledge on FinTech success scattered in the scientific literature on distinct FinTech business model archetypes. Hence, the first iteration of our taxonomy-based process implements a conceptual-to-empirical approach, followed by six empirical-to-conceptual paths in the form of an iterative content analysis of research articles related to FinTech success that have been identified in the previous section. All iterations were performed manually in line with the taxonomic process and ending conditions (Nickerson et al., 2013). Below, we outline the deductive approach to conceptualize our preliminary taxonomic structure and the iteration paths carried out during the development process of our final taxonomic structure of FinTech SFs:

### 1st iteration

The first iteration of our taxonomy-based process implemented a conceptual-to-empirical path to deductively conceptualize a preliminary taxonomic structure by subtracting dimensions and characteristics from the scientific literature on three axes connected to our meta-characteristic: business model research, SF research, and the FinTech business model taxonomy of Eickhoff et al. (2017). So far, this taxonomy offers the most comprehensive insight into the entire landscape of FinTechs and their corresponding business models. Therefore, we adopted the FinTech business model archetypes identified by Eickhoff et al. (2017) as a base for our analysis to draw the dimensions of (i) product/service offering, (ii) dominant technology component, (iii) delivery channel, (v) target customer, (v) revenue stream, and (vi) value proposition. These dimensions have their theoretical foundations in the knowledge field of business models (e.g., Alt & Zimmermann, 2001; Osterwalder et al., 2005), but their relevance in the FinTech context has been validated by Eickhoff et al. (2017). In line with Thompson et al. (2018), other aspects such as strategy elements, operational approaches, resources, and competitive capabilities are also success components. We, therefore, deduced the dimensions (vii) strategic factors from Chesbrough and Rosenbloom (2002), (viii) operational factors from the seminal business model research publications of Osterwalder et al. (2005) and Zott et al. (2011), (ix) cost factors and (x) stakeholder factors from Osterwalder and Pigneur (2010) along with Al-Debei and Avison (2010); as well as the dimension of (xi) input resources from Hedman and Kalling (2003). We chose the latter dimension under the premise that the efficient utilization of input resources like human capital as well as data and information is especially relevant within the FinTech industry. Furthermore, the technology level used can be seen as an industry-specific SF (Alt & Zimmermann, 2001), especially in industries like FinTech, where business innovation is enabled by IS (Eickhoff et al., 2017). DeLone and McLean (2003) state that effective IS success depends on technical and semantic success. These, in turn, produce net benefits that impact the use of the service/information produced and the user’s attitude toward the system characteristics. Based on this, we derive from the literature on IS success (e.g., Ain et al., 2019; DeLone & McLean, 2003; Petter et al., 2013) the dimensions (xii) user factors and (xiii) technological factors. Lastly, to consider external factors that can influence the competitive success of FinTechs (David & David, 2017; Rockart, 1979), e.g., to gain and maintain a competitive advantage, we incorporate the dimension (xiv) environmental factors. As a result, we deducted a set of 14 dimensions in this iteration. To broaden these dimensions, we compounded further related characteristics from our knowledge and experience accumulated through academic conferences, scientific publications, and the analysis of diverse digital transformation case studies in the financial sector. Table 1 presents the deductive dimensions and characteristics as well as primary scientific papers used as their conceptual bases.

**Table 1:** Conceptual taxonomic structure of potentially relevant FinTech success factors

Dimensions Di	Characteristics Ci,j	Conceptual base
D1 Product/service offering	D1 = {C1,1 Brokerage, C1,2 Credit/lending, C1,3 Currency exchange, C1,4 Current account, C1,5 Device, C1,6 Financial education, C1,7 Financing, C1,8 Fraud prevention, C1,9 Information aggregation, C1,10 Investments, C1,11 Payment service, C1,12 Personal assistant, C1,13 User identification}	Eickhoff et al. (2017)
D2 Dominant technology component	D2 = {C2,1 Peer-to-peer technology, C2,2 Digital platform, C2,3 Analytics, C2,4 Online marketplace, C2,5 Database, C2,6 Transaction processing system (TPS)}	Alt and Zimmermann (2001); Eickhoff et al. (2017)
D3 Delivery channel	D3 = {C3,1 API, C3,2 Mobile applications, C3,3 Physical, C3,4 WWW, C3,5 WWW + App, C3,6 Instant message, C3,7 Peer-to-peer network/Marketplace}	Eickhoff et al., (2017); Osterwalder et al., (2005)
D4 Target customer	D4 = {C4,1 B2B, C4,2 B2C, C4,3 B2B, B2C, C4,4 P2P}	Eickhoff et al., (2017); Osterwalder et al., (2005)
D5 Revenue stream	D5 = {C5,1 Kickback, C5,2 Pay per use, C5,3 Revenue share, C5,4 Sales, C5,5 Subscription, C5,6 Free, C5,7 Hybrid, C5,8 Commission rebate, C5,9 Bonus/reward system}	Eickhoff et al., (2017); Osterwalder et al., (2005)
D_6_ Strategic factors	D_6_ = {C_6,1_ Corporate plan, C_6,2_ Competitive plan, C_6,3_ Brand-building, C_6,4_ Marketing plan, C_6,5_ Growth plan, C_6,6_ Innovation culture, C_6,7_ Operational design}	Chesbrough and Rosenbloom (2002); Thompson et al. (2018)
D_7_ Operational factors	D_7_ = {C_7,1_ Operational structure, C_7,2_ Operational processes, C_7,3_ Operational policies, C_7,4_ Operational infrastructure, C_7,5_ Operational resources, C_7,6_ Quality assurances, C_7,7_ Management skills}	Osterwalder et al. (2005); Thompson et al. (2018); Zott et al. (2011)
D_8_ Input resources	D_8_ = {C_8,1_ Data and information, C_8,2_ Industry know-how, C_8,3_ Human capital, C_8,4_ Machinery/equipment, C_8,5_ Financial capital}	Hedman and Kalling (2003); Thompson et al. (2018)
D_9_ User factors	D_9_ = {C_9,1_ User demographics, C_9,2_ Consumption patterns, C_9,3_ User centricity, C_9,4_ User knowledge, C_9,5_ User satisfaction, C_9,6_ User trust, C_9,7_ Usability, C_9,8_ User perceived quality, C_9,9_ User perceived time effort, C_9,10_ Cost attractiveness}	Osterwalder et al., (2005); Petter et al. (2013); Thompson et al. (2018)
D_10_ Technological factors	D_10_ = {C_10,1_ Technology upgradation, C_10,2_ Technological capabilities, C_10,3_ Technology adoption, C_10,4_ Technology profitability, C_10,5_ Data security, C_10,6_ Security risks, C_10,7_ IT planning, C_10,8_ Efficiency, C_10,9_ Technology trends}	Alt and Zimmermann (2001); DeLone and McLean (2003); Petter et al. (2013)
D_11_ Value proposition	D_11_ = {C_11,1_ Automation, C_11,2_ Collaboration, C_11,3_ Convenience/usability, C_11,4_ Customization, C_11,5_ Financial risk, C_11,6_ Financial insight, C_11,7_ Intermediation, C_11,8_ Security, C_11,9_ Transparency, C_11,10_ Unification/consolidation, C_11,11_ Monetary, C_11,12_ Decision support}	Eickhoff et al. (2017); Osterwalder et al. (2005)
D_12_ Cost factors	D_12_ = { C_12,1_ Marketing cost, C_12,2_ Infrastructure cost, C_12,3_ Operational cost}	Al-Debei and Avison (2010); Osterwalder et al. (2005)
D_13_ Stakeholder factors	D_13_ = {C_13,1_ Specialized talent/knowledge workers sourcing, C_13,2_ Stakeholder performance expectations, C_13,3_ Strategic networks and alliances}	Al-Debei and Avison (2010); Osterwalder et al. (2005)
D_14_ Environmental factors	D_14_ = {C_14,1_ Global market conditions, C_14,2_ Capital market conditions, C_14,3_ economic infrastructure, C_14,4_ External regulations, C_14,5_ Government support, C_14,6_ industry rivalry}	Alt and Zimmermann (2001); Hedman and Kalling (2003)

### 2nd–7th iteration

**Fig. 3:**
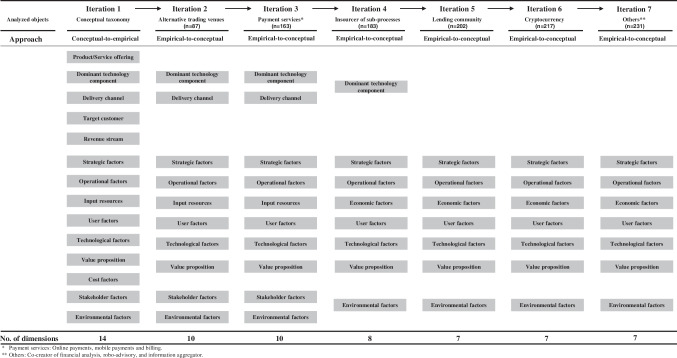
Dimensions development process

In six inductive empirical-to-conceptual iterations, we examined all relevant research articles and identified the factors related to FinTech success. In these seven iterations, we carefully read every paper, i.e., the data, that we found per archetype, and checked the paper results for relevant SFs and related content. Consequentially, we checked the scientific articles for relevancy. Possible SFs mentioned by the authors of the papers must be directly connected to the respective archetype. If SFs were identified, we considered them for further analysis. We used MAXQDA 2020 and Microsoft Excel as supporting software for this qualitative content analysis. Subsequentially, thereby classified the found factors into the deductive initial taxonomy to refine it, e.g., by removing non-descriptive elements. An overview of the examined objects (scientific articles) and the dimensions during the taxonomy development process is provided in Fig. 3. A detailed description of the applied steps at the characteristic and dimensional level and an overview of the subjective and objective ending conditions fulfilled in each iteration are provided in the appendix, Tables 7 and 8, respectively. Since we did not modify the dimensions and characteristics in the seventh iteration and the objective and subjective ending conditions were fulfilled, we ended the taxonomy development process.

With our taxonomic process, we could classify potentially determinant FinTech SFs from academic literature. We observe that FinTech SFs, identified by researchers, can be classified and categorized into seven dimensions: (i) strategic factors, (ii) operational factors, (iii) technological factors, (iv) value proposition, (v) user factors, (vi) economic factors, and (vii) environmental factors. These dimensions are composed of 31 characteristics across all FinTech business model archetypes, as shown in Table 2. For example, D_1_ (the strategic factor dimension) contains four characteristics, C_1,1_ to C_1,4_, that characterize the strategic factors in more detail. Here, a strategic vision and action plan to achieve the overall goals of the FinTech venture (corporate plan) or a strategic allocation of resources to support regular business routines or the development of technological infrastructure (operational design) can be named.

**Table 2:** Final taxonomic structure of FinTech success factors

Dimensions D_i_	Characteristics C_i,j_		
D_1_ Strategic factors	C_1,1_ Corporate plan	C_1,2_ Operational design	
	C_1,3_ Competitive plan	C_1,4_ Marketing plan	
D_2_ Operational factors	C_2,1_ Competency-based human resources	C_2,2_ Strategic networks and alliances	
	C_2,3_ Operational alignment	C_2,4_ Cost–benefit dynamic of the innovation	C_2,5_ Efficiency
D_3_ Technological factors	C_3,1_ Technology integration	C_3,2_ Technology adoption	
	C_3,3_ Security, privacy, and transparency	C_3,4_ Environmental sustainability	C_3,5_ Ethical issues
D_4_ Value proposition	C_4,1_ Convenience/usability	C_4,2_ Customization	C_4,3_ Intermediation
	C_4,4_ Monetary	C_4,5_ Disintermediation	C_4,6_ Decision support
D_5_ User factors	C_5,1_ User socio-economic characteristics	C_5,2_ User centricity	C_5,3_ User trust
	C_5,4_ User-perceived quality	C_5,5_ Cost attractiveness	C_5,6_ Ease of use
D_6_ Economic factors	C_6,1_ Financial capital	C_6,2_ Cost structure	
D_7_ Environmental factors	C_7,1_ Industry rivalry	C_7,2_ Market conditions	C_7,3_ Regulation

As a supplement, we present definitions for all taxonomy dimensions and characteristics in Table 3. Definitions are based on literature used for our preliminary taxonomic structure in the first iteration and have been tailored for the FinTech context.

**Table 3:** Definitions for all taxonomy dimensions and characteristics

Dimension/characteristic	Definition
D1 Strategic factors	Describe strategic factors that are relevant for FinTech venture success
C1,1 Corporate plan	Strategic vision and action plan to achieve the overall goals of the FinTech venture
C1,2 Operational design	Strategic allocation of resources to support regular business routines or the development of technological infrastructure
C1,3 Competitive plan	Strategic plan to foster the development of relative strengths and advantages of a FinTech venture over its competitors in the industry
C1,4 Marketing plan	Go-to-market strategies to achieve a market-oriented competitive advantage through advertising efforts
D2 Operational factors	Describe operational factors that are relevant for FinTech venture success
C2,1 Competency-based human resources	Ability to internally develop specialized human resources with comprehensive industry know-how (i.e., domain experts) or the capacity to outsource and manage specialized talent/knowledge
C2,2 Strategic networks and alliances	Agreement between two or more parties to pursue a set of shared objectives while remaining independent organizations
C2,3 Operational alignment	The extent to which processes and operational policies align to create the environment that enables companies to reach their goals
C2,4 Cost–benefit dynamic of the innovation	Balance of the practical or economic benefit of a new product or service in relation to its (technology) costs
C2,5 Efficiency	Ability to offer products and services with a minimum of effort, expense, or waste
D3 Technological factors	Describe technological factors that are relevant for FinTech venture success
C3,1 Technology integration	Ability to take advantage of diffused knowledge on disruptive innovations to incorporate new technical resources
C3,2 Technology adoption	Competence to integrate and upgrade new or existing technologies to make innovative products and services
C3,3 Security, privacy, and transparency	Ability to provide products and services without compromising customer privacy, in accordance with security standards, and transparently communicate issues
C3,4 Environmental sustainability	Capacity to achieve financial and non-financial goals in accordance with environmental sustainability principles
C3,5 Ethical issues	Ability to recognize, discuss, communicate, and find solutions to ethical issues
D4 Value proposition	Describe the central values the company delivers to the market to archive venture success
C4,1 Convenience/usability	Capacity to deliver a useful product or service easily or provide access to a market for the customer in a practical way
C4,2 Customization	Capability to provide a product or service in a customizable and personalized way to the customer
C4,3 Intermediation	Competence to mediate the direct interaction and collaborative agreements of multiple affiliated or anonymous parties
C4,4 Monetary	Capability to provide liquidity to customers, e.g., through loans
C4,5 Disintermediation	Ability to provide disintermediation services, e.g., by using Blockchain-based business models
C4,6 Decision support	Capacity to provide support on financial decision-making
D5 User factors	Describe user-specific issues that are relevant for FinTech venture success
C5,1 User socio-economic characteristics	Characteristics of consumer populations that are important to the development and commercialization of products and services
C5,2 User centricity	Alignment of all business activities to enhance the customer experience
C5,3 User trust	The extent to which customers rely on a company’s products or services and their tendency to select one brand over the competition
C5,4 User-perceived quality	The notion of quality that a customer experiences about a product or service
C5,5 Cost attractiveness	Ability to offer a product or service at an appropriate price without sacrificing quality
C5,6 Ease of use	Ability to offer a product or service which is easy to use by its intended users
D6 Economic factors	Describes what economic issues are relevant for FinTech venture success
C5,1 Financial capital	Economic resources are needed to offer products and services
C5,2 Cost structure	Types and relative proportions of fixed and variable costs of a company
D7 Environmental factors	Describe the macroeconomic characteristics of the market
C7,1 Industry rivalry	Ability to endure in a competitive industry sector
C7,2 Market conditions	Global market conditions manifested in the capital market have an impact on, e.g., commodity pricing, trading volume, and willingness of market participants to invest
C7,3 Regulation	The legislation imposed by a government to regulate and modify economic behaviors

After ending our taxonomic process, we took a more holistic view of all archetypes, SFs, and academic articles found. Using this taxonomic structure, we identified the most predominant characteristic within each dimension at the archetype level for the eight other archetypes and visualized them in Table 4. We did not find any literature on the archetypes “financial market intermediary” and “information extractor.” These two archetypes are, therefore, not listed in the table. To illustrate the degree of generality of the factors, i.e., scope in theory for analysis, the relative frequency distribution of the FinTech SFs is shown. Several descriptive observations and highlights can be named from our literature search results presented:

**Table 4:**
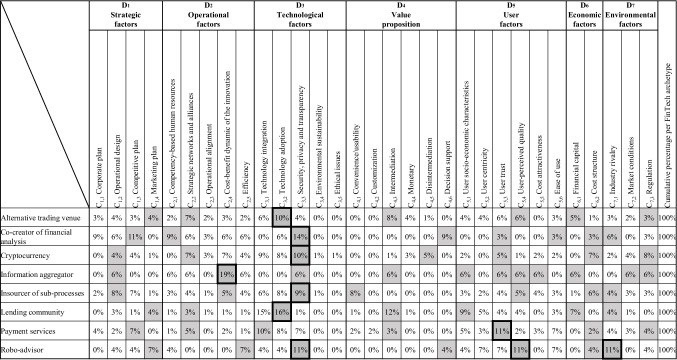
Relative frequency distribution of relevant fintech success factors

Bold black border means most relevant success factors per FinTech business model archetype; Gray box means most relevant success factors per dimension; no literature was found for the business model archetypes “ Financial markets intermediary and “Information extractor”

We obtained these distributions by dividing the frequency of the identified SFs per characteristic by the total data set of factors identified per the FinTech business model archetype. We then multiply the former result by 100 to obtain the relative percentage range corresponding to each characteristic (Freund et al., 2010). The bold black borders in Table 4 indicate the most relevant SF per FinTech business model archetype. At the same time, the gray highlighted boxes denote which characteristic is the most relevant SF per dimension according to our taxonomy-based analysis. For six of eight business model archetypes, we can observe that technological factors play an important role in their success. More precisely, “security, privacy, and transparency” have been investigated as the most relevant SF for the “co-creator of financial analysis (14%),” “cryptocurrency (10%),” “insourcer of sub-processes (9%),” and “robo-advisor (11%)” archetypes. “Technological adoption” were investigated and located as SF in the past for “alternative trading venues (10%)” and “lending communities (16%).” In the case of “lending communities,” we found that “technological adoption” was the second highest SF for a specific archetype based on our dataset. In the context of our study, we understand “technology adoption” as the competence to integrate and upgrade new or existing technologies to make innovative products and services (see Table 3). Likewise, the characteristics of “user trust” from the dimension of “user factors” and “technology adoption” were identified as the most relevant SF for the archetypes “payment services (11%),” and “alternative trading venues (10%)” respectively. Also, especially “information aggregators” have the operational factor of the “cost–benefit dynamic of the innovation” as a crucial SF (19%) and the highest relative frequency found. “Cost–benefit dynamic of the innovation” means a balance of the practical or economic benefit of a new product or service about its (technology) costs. To sum up, we can observe a clear tendency of most SFs to be located in the “technological factors” dimension. Furthermore, Table 4 shows that past literature provides a somewhat consensus on the SFs for “information aggregator” and “lending communities.” In contrast, “robo-advisors,” for example, are influenced by a more diverse set of SFs, represented through three bold black boxes

## Case-based taxonomy validation and usefulness with examples from the FinTech industry and interviews

We va﻿lidate and compare our taxonomy to demonstrate its usefulness and applicability with real-world examples from the FinTech industry. This case-based taxonomy validation is useful to show the ex-post applicability of identified SFs from the literature concerning real-world objects, i.e., FinTechs for each archetype (Kundisch et al., 2021). We identified FinTechs for each archetype within the Crunchbase database (Cruchbase, 2022a) and used additional information from Google News about the respective FinTech concerning our identified SF. We looked for so-called Series C FinTechs, which are more established companies in later funding rounds, of around ten or more Million US$. Focusing on Series C FinTechs, we argue that FinTechs in these stages of their business life-cycle show success with their business model and underlying activities. As a result, they serve as appropriate cases and examples to compare with our identified SF in Table 4.

### Alternative trading venues

PeerStreet, an example of a successful FinTech within the “alternative trading venues” archetype, is a real estate crowdfunding platform founded in the USA. PeerStreet pursues the goal of providing democratic access to investments in real estate debt through advanced algorithms and big data analytics (Businesswire, 2022a, b; Globalratings, 2022). For this FinTech archetype, the most important identified SF is “C_3,2_ technology adoption”; hence, at the practical level, this SF is reflected in terms of the capability of PeerStreet to penetrate the market and reach a critical mass of customers by automating, simplifying, and speeding up the process of underwriting real estate loans. To achieve a profitable cash flow in the long-term, PeerStreet needs to steer its resources toward reaching a critical mass customer or expanding its customer base by targeting a larger market, enabling the company to self-finance and further scale up its business.

### Co-creator of financial analysis

“C_3,3_ security, privacy, and transparency” is critical for the success of FinTechs within the archetype “co-creator of financial analysis” (Findbiometrics, 2022). Biocatch is an example of a successful financial analytics Fintech, which leverages machine learning and behavioral biometrics to offer fraud protection and digital security solutions (Findbiometrics, 2022). The business model of Biocatch relies upon digital behavior datasets to analyze the physical and cognitive behavior of financial service users digitally. This, in turn, can lead to security, privacy, and transparency concerns among end-users concerning biometric solutions. To offset these concerns, the digital behavior databases used by Biocatch are not based on personally identifiable information, and their technological solutions are seamlessly integrated into the financial processes of financial institutions obliged to comply with privacy and security laws (The Paypers, 2022).

### Cryptocurrency

For the FinTechs within the archetype cryptocurrency, “C_3,3_ security, privacy, and transparency” was also identified as the most important SF. CoinDCX is an example of a successful crypto-trading FinTech that aims to democratize investments through cryptocurrency-based financial services (Cruchbase 2022b). CoinDCX is India's first crypto-trading FinTech unicorn (a startup company valued at over $1 billion). CoinDCX strives to use a security-first approach to consolidate its position in the market as a differentiating factor from the competition. It seeks to become India’s most secure cryptocurrency exchange by implementing information security strategies, governance processes, and data protection programs (Livemint, 2022).

### Information aggregator

MX Technologies, an example of a successful FinTech within the information aggregator archetype, is a data aggregation platform established in the USA. MX Technologies aggregates, analyze, and processes unstructured financial data using artificial intelligence and machine learning to leverage customer-centric banking applications and solutions (Cruchbase, 2022c). An important SF for FinTechs belonging to the information aggregator archetype is the “C_2,4_ cost–benefit dynamic of the innovation.” MX Technologies contributes to this factor by offering unique and specialized value propositions that enable its customers (e.g., financial institutions and digital banking providers) to improve their level of business intelligence. Customers are thereby enabled to make data-driven decisions based on trends in customer behavior, reduce costs derived from broken business processes, and accelerate their ability to pursue product and service innovations (Prnewswire, 2022). The cost–benefit dynamic of MX Technologies allows its customers to benefit from the acquisition of ready-to-use banking applications and solutions without having to make costly investments in developing such solutions in-house. At the same time, MX Technologies also benefits from lock-in effects.

### Financial markets intermediary

Concerning the archetype financial markets intermediary, no relevant scientific literature has been found on the SFs of this type of FinTechs. Nonetheless, we analyze the characteristics of a German social trading FinTech named ayondo as an example of a FinTech within this archetype that, despite having maintained a run of success, ended up on the verge of failing due to regulatory changes. Although ayondo established strategic networks and alliances with top-tier banks, it aimed to be a financial services provider, targeting the end-customer market (Ayondo, 2022; Startupticker, 2022). To increase the scope of its market, ayondo opted for a competitive plan involving global branding, extending its operation to the UK (i.e., ayondo markets Ltd in London) and Asia (i.e., ayondo Ltd in Singapore). However, a FinTech company that operates in more than one country faces additional regulatory obligations due to different legal forms and restrictions. In Germany, the business model of ayondo was impacted by the enactment of new regulations in terms of privacy and security laws, such as the General Data Protection Regulation (GDPR). Furthermore, the London-based sister company was not licensed to continue business operations after the UK exit from the European Union. Because of the regulatory changes imposed by European and British regulators, ayondo suffered continuous operating losses and ultimately faced a working capital deficiency, which led to ayondo markets Ltd filing for insolvency and delisting ayondo Ltd from the Singapore Exchange (Ayondo, 2022; Financefeeds, 2022). Ayondo portfolio management GmbH has received an asset management license and strives to continue its business activities.

### Information extractor

We did not find relevant literature that deals with SFs about information extractor FinTechs. However, we analyzed the characteristics of M2P Fintech as an example of a successful information extractor. M2P Fintech provides custom API platforms for banking and payment products (M2P FinTech, 2022). Founded in India, M2P Fintech is in a unique position and faces little to no competition in Asian countries (Techcrunch, 2022). The “C_1,3_ competitive plan” here has played a role as an SF since M2P Fintech started and expanded in the Asian regions where competition is low for API platforms.

### Insourcer of sub-processes

As an example of a successful insourcer of sub-processes, Juniper Square is a real estate investment management provider in the USA. Since “C_3,3_ security, privacy and transparency” was identified as the most SF for this archetype, Juniper became very successful with SOC-2 compliance, a certification for data protection and trustworthy customer data usage. Furthermore, high-security standards, combined with the “C_4,1_ convenience/usability” of the service, play an important role in the success of Juniper (Businesswire, 2022a, b).

### Lending community

“C_3,2_ technology adoption” is the most important SF for FinTechs within the archetype lending community. ZestMoney is an Indian consumer lending company that connects customers with their lending partners (ZestMoney, 2022). Delivered through a mobile application that is supported by artificial intelligence, ZestMoney shows the competence to integrate and upgrade new or existing technologies, i.e., mobile applications with AI, to make innovative products and services for the customer (Entrepreneur, 2022).

### Payment services

Payment services must provide a high degree of “C_5,3_ User trust” to be successful. CURVE is a payment service founded in the UK. CURVE is an adequate example of a FinTech that focuses on security issues. For example, details of credit cards at CURVE are not stored on the user’s mobile device. Fraudulent behavior can thereby be avoided (Curve, 2022). This increases user trust and raises the tendency of the customers to use their services continuously.

### Robo-advisory

For robo-advisors, we identified three SFs that play an important role in the survival of FinTechs, namely “C_3,3_ security, privacy and transparency,” “C_5,4_ user-perceived quality,” and “C_7,1_ industry rivalry.” Albert, a successful US-based robo-advisory service, delivers automated advisory for its customers based on their preferences (Albert, 2022). Albert built up high privacy standards through an A-rating by the Better Business Bureau, focusing on factors like customer complaint histories. While showing no lawsuits or public scandals, Albert has been very competitive within the robo-advisor market (Americanbanker, 2022; Businessinsider, 2022).

We also discussed our findings for the usefulness of our taxonomy-based analysis with two individuals from the FinTech sector. Interview partners were located within the author’s networks and invited through e-mail. Both interviews lasted around 40 min and were held in German. They were conducted online and not recorded. Interview notes served then as primary data. We focused on evaluating our results and findings regarding usefulness and appropriateness for relevant target user groups within the FinTech ecosystem. Guidance for taxonomy evaluation proposed by Kundisch et al. (2021) was used. We interviewed one account executive from a FinTech with around 1000 employees (I1). Following the archetypes provided in this study, this FinTech offers “payment services” and “information aggregator” products. Another interview was held with a senior researcher from the FinTech sector, who is now the technical director of a start-up in the Internet of Things sector (I2). This researcher was not involved in the taxonomy development process before to be independent in his/her statements, as suggested by Kundisch et al. (2021).

Generally, both interview partners liked the content and structure of the results provided. Here, the view on different archetypes within the FinTech sector was mentioned (I1). The expert from the interviewed FinTech was somewhat able to recognize SFs from its own business, e.g., “financial capital.” As possible main target groups for this study, banks, e.g., FinTech hubs and consultants, and FinTechs in the first stage of their life-cycle are named. However, the SFs provided should be treated as supplementary information and a knowledge base for a possible reallocation of business activities (I1; I2). Stakeholders like venture capitalists are followed by their own experiences and gut feelings (also mentioned by Werth et al., (2019)). Nevertheless, this target group can use it as a knowledge base. I2 marked those venture capitalists would use this information as a second-order information base within a due diligence process after the first own screening of potential FinTechs to invest in. I1 said that the dimensions provided are interesting, but FinTechs must prioritize their importance themselves. In this case, “value proposition” and “user factors” are most important, then “environmental factors” are considered. I2 also confirms this. I2 argued that the information provided in Table 4 could lead to meaningful future research on different FinTech archetypes. However, future research inquiries should consider and compare the literature on SFs of start-ups in general, e.g., team compositions, with the information provided in this study toward more detailed business model configurations.

## Discussion of academic literature on success factors and real-world FinTechs per dimension

### D_1_ Strategic factors

Our analysis shows that the success of FinTech cloud-based or analytics-based business models (e.g., “insourcer of sub-processes” and “information aggregator”) relies to a large extent on the *design of operational reliability plans* to counteract shortcomings in their systems such as overflow, network, and software failures (e.g., Buyya et al., 2018). Alternatively, other FinTech business model archetypes such as “alternative trading venues,” “lending community,” and “robo-advisor” rely on *social media marketing plans* as drivers for their business models. For example, with social media marketing, FinTechs can build a digital community of supporters through digital audience-targeting strategies. Additionally, they can better shape the subjective norm prevailing in social media networks about their knowledge/awareness, perceptions, attitudes, and beliefs of the users (e.g., Clauss et al., 2020; Crosetto & Regner, 2018). This allows the FinTech business model archetypes to obtain approval, optimize campaign/service awareness, and maintain control over the communication and the disclosed information to the target digital community (Crosetto & Regner, 2018; Gerber & Hui, 2013). We see this issue, especially at M2P FinTech, which has a competitive marketing plan supported through social media activities. While social media marketing strategies may contribute further to the success of these social media-oriented FinTech archetypes, biased information and quality signals, e.g., the presence of exaggeration bias, have a detrimental effect on their potential success (Momtaz, 2020).

On the other hand, the FinTech business model of “co-creator of financial analysis” and “payment services” are driven by *plans to build competitive intelligence* as relevant strategic factors for their success. In the first case, the “co-creator of financial analysis” depends on the development of competitive plans based on the strategic fostering of big data analytics capabilities to provide business value to customers through the analysis of both structured and unstructured data and the automation of routine analytics functions (e.g., Mikalef et al., 2019; Richins et al., 2017). In the second case, the success of the “payment services” is centered on deploying competitive plans to achieve the widest possible international customer base and greater control over the network configuration (e.g., Rukanova et al., 2020). Nonetheless, *corporate plans* (e.g., strategic vision/orientation) appear less relevant for FinTech success than other strategic factors. Yet, this strategic factor can affect, for example, the perception of risk and uncertainty of FinTechs within the business model archetypes “alternative trading venues,” “co-creator of financial analysis,” “insourcer of sub-processes,” and “payment services” (e.g., Kauffman et al., 2018). In contrast, based on our case-based validation, we found that this corporate plan seems relevant for the success of the archetype “information extractor,” as identified for the FinTech M2P.

### D_2_ Operational factors

The success of FinTech business models with the archetypes “alternative trading venues,” “cryptocurrency,” “lending community,” and “payment services” depend mainly on the *interplay of strategic networks and alliances* to achieve network effects (e.g., Crosetto & Regner, 2018). These can contribute to decreasing costs of entrepreneurial effort (e.g., product/services innovations or advertising) of crowdfunding, lending, and ICO campaigns, through the establishment of a community of supporters to prompt emotional commitment and long-term interaction in the form of, e.g., information/learning exchanges, recommendations, feedback on products or services, and word-of-mouth propaganda (e.g., Kher et al., 2020; Schückes and Gutmann, 2020). Additionally, the forge of a strategic alliance with key influencers, such as renowned venture capitalists, private investors, insurers, or universities, can be used as business tools to transmit quality signals to potential investors. This offsets information asymmetries and reduces agency costs associated with sources of uncertainty such as the reputation of the FinTech entrepreneur or the nature of the FinTech product or service offering itself, among others (e.g., Kher et al., 2020; Momtaz, 2020). In the case of FinTech business models, “payment services,” the strategic alliances contributing to FinTech success mainly integrate providers’ services and constitute partnerships with banks and existing mobile banking applications. These improve the access of FinTechs to the existing payments network and enable, for example, interbank transfers (e.g., Ondrus et al., 2009).

In contrast, the success of “information aggregator” and “insourcer of sub-processes” FinTechs was frequently associated with the *cost–benefit dynamic of the innovation*. This implies that in FinTech business models with low entry barriers or with high levels of substitute competition, the realization of economic efficiency and a positive relative advantage, e.g., by a competent balance between the revenue model and transaction or switching costs are important (e.g., Thitimajshima et al., 2018; Trenz et al., 2013). As shown in the case studies section, for FinTech business models with low levels of substitute competition, it is particularly important to provide innovative and immediate responses to problems that customers would only solve on their own with high amounts of investment of time or money.

Meanwhile, for their continued success, “co-creator of financial analysis” FinTechs must have sufficient *competency-based human resources* comprising domain experts with advanced statistical knowledge, programming, analytical, and communication skills to communicate their results to non-technical executives (Liu et al., 2018). Also, the users of these FinTech services must possess relevant technical skills and domain knowledge to use the embedded business value (Mikalef et al., 2019).

### D_3_ Technological factors

Our analysis shows that scientific research is mostly focused on the analysis of security, privacy, and transparency, as well as the adoption of the dominant technology enabling a respective FinTech business model. Based on our analysis, the technological factors *security, privacy, and transparency* are relevant for the success of the FinTech business model archetypes “co-creator of financial analysis,” “cryptocurrency,” “information aggregator,” “insourcer of sub-processes,” and “robo-advisor.” Connected to the validation of “insourcer of sub-processes” and “robo-advisor,” we can confirm these statements. Privacy and security concerns related to the intended privacy, more specifically the transaction anonymity of blockchain-based business models, are associated with a potential risk of being used to perform illegal activities such as money laundering (e.g., Cousins et al., 2019). The pseudonymization of blockchain transactions is an appealing factor for using cryptocurrencies as the number of anonymity techniques increases, e.g., through centralized and decentralized mixing or ring signatures. Therefore, a better balance between privacy and the capacity to trace illegal transactions through digital forensic techniques is fundamental for the sustainable and ethical use of blockchain-based FinTechs (Cousins et al., 2019; Genkin et al., 2018). These FinTech business models also suffer security threats such as cyber-attacks, which can increase skepticism and restrain potential customers and governments. However, as shown in the case studies section, FinTechs with blockchain-based business models should turn their efforts to manage information security and privacy risks into a differentiating factor to gain a competitive advantage.

Regarding cloud-based FinTech business models (e.g., “insourcer of sub-processes”), in particular, the mitigation of security and control issues related to public clouds (see, e.g., Garrison et al., 2012) and mobile cloud storage augmentation systems (e.g., Zhou & Buyya, 2018) is relevant for their success. Furthermore, their aim should be to improve the privacy aspects in cloud-based applications (e.g., data confidentiality and protection) by using encryption, cloud indexing, data splitting, or secure enclaves (Buyya et al., 2018). For the “information aggregator” and “robo-advisor” FinTech business models, the level of data privacy and transparency concerning the underlying technological structures, operational processes, and pricing models are highly relevant for success (e.g., Jung et al., 2018; O’Reilly & Finnegan, 2010).

Likewise, the *technology adoption* factor is related to the users’ intrinsic motivations, such as ideological or philosophical motivations, e.g., the “personal enthusiasm for the business model/idea” or the “personal enthusiasm for the technology” (Fisch et al., 2019), is relevant for the success of the FinTech business model archetypes of “alternative trading venue” and “lending community.” As noted in the case studies section, FinTechs targeting the end customer market must understand the knowledge of users as well as the affective and behavioral factors that form the users’ decision to adopt their technologies. Also, FinTechs targeting the end customer market must reach a critical mass of customers to achieve a sustainable cash flow in the long term. Therefore, successful “alternative trading venue” and “lending community” FinTechs need to understand the factors that form the customers’ perceptions, beliefs, and attitudes concerning adopting their technologies. On the other hand, the *technology integration* factor related to the level of integration of new technologies such as biometrics, quick response (QR) code, and near field communication (NFC) payments is especially relevant for the success of the FinTech business model archetype “payment services” (e.g., Ondrus et al., 2009; Singh & Sinha, 2020). Lastly, characteristics such as *environmental sustainability* (i.e., energy-intensive consumption) and *ethical issues* have an increasing impact on the sustainability of cloud and blockchain-based FinTech business models, e.g., “cryptocurrency” and “insourcer of sub-processes” (Buyya et al., 2018).

### D_4_ Value proposition

Our analysis identified the prospective value benefits of *intermediation* (concerning “alternative trading venues,” “information aggregator,” “lending community,” and “payment services”); *convenience/usability* (regarding “insourcer of sub-processes”); *disintermediation* (to “cryptocurrency”), and *decision support* (regarding “co-creator of financial analysis”) as the value propositions mostly associated with success. Especially for the archetype “insourcer of sub-processes,” we found that *convenience/usability* was perceived as important for the success of FinTech Zest Money. The value proposition of *intermediation* contributes to the success of the FinTechs as mentioned earlier. It is enabled by the creation of business value, for example, through online platforms to mediate the direct interaction and collaborative agreements of multiple affiliated or anonymous parties in terms of capital or data exchange (see, e.g., Kleinert et al., 2020; Koch & Siering, 2019; Mikalef et al., 2019). On the other hand, the value proposition *disintermediation* in FinTech furthers the success of primarily blockchain-based FinTech business models through philosophical factors, for example, increased libertarianism, the democratization of financial services, and financial inclusion.

### D_5_ User factors

Our analysis identified the relevant user factors that form the basis for assessment and the decision of potential users to choose the product or service offerings provided by FinTechs. *User trust* regarding “co-creator of financial analysis,” “cryptocurrency,” “information aggregator,” and “payment services,” given the association of the notion of trust to the perceived risks (e.g., Hong & Cha, 2013; Mikalef et al., 2019). *User-perceived quality* (e.g., reputation) concerning “alternative trading venue,” “information aggregator,” “insourcer of sub-processes,” and “robo-advisor” (e.g., Kleinert et al., 2020). *User socio-economic characteristics,* such as cultural or geographical similarities, regarding “information aggregator” and “lending community” (e.g., Chen et al., 2016); and *cost attractiveness* in connection with “information aggregator”; and *ease of use*, relating to “co-creator of financial analysis” (e.g., Thitimajshima et al., 2018). Our analysis shows that while *user-centricity* is less important than the abovementioned user factors, it is relevant for most FinTech business model archetypes, i.e., “alternative trading venue,” “insourcer of sub-processes,” “lending community,” “payment services,” and “robo-advisor” (e.g., Buyya et al., 2018). In contrast, our taxonomy validation identified user trust and quality as SF accelerating funding for the real-world FinTech example.

### D_6_ Economic factors

The identified relevant SFs include the potential for raising, lending, or investing capital beyond the traditional methods with low entry barriers (e.g., Cummings et al., 2020; Momtaz, 2020; Schückes & Gutmann, 2020), as well as the *cost structures* of FinTechs (e.g., Buyya et al., 2018). These provide an entrance platform for FinTech through product, service, or cost differentiation. In this context, the economic factor of *financial capital* is relevant for the success of the FinTech business model archetypes “alternative trading venue,” “information aggregator,” and “lending community.” However, the cost structure is relevant for the success of “co-creator of financial analysis,” “cryptocurrency,” “insourcer of sub-processes,” “payment services,” and “robo-advisor” FinTechs. A combination of elements can enhance the potential for raising, lending, or investing venture capital through FinTechs, e.g., signal quality indicators such as prior financing success (e.g., Kleinert et al., 2020) or participation in accelerators programs (e.g., Ralcheva & Roosenboom, 2019). Conversely, high costs attributed to hardware infrastructure investments, e.g., to enhance big data analytics capabilities (e.g., Mikalef et al., 2019) or for cryptocurrency mining purposes, as well as high operational energy consumption due to the use of non-cost-efficient network elements like cloud systems (e.g., Buyya et al., 2018) can negatively affect the sustainable cost differentiation of FinTechs in relation to their competitors.

### D_7_ Environmental factors

The* regulation* was identified as a relevant SF for the FinTech business model archetypes “alternative trading venues,” “cryptocurrency,” “information aggregator,” and “payment services” because of its cross-cutting scope to outline and delimit their fields of application, e.g., through tax and trading conditions (e.g., Cousins et al., 2019; Kher et al., 2020). Moreover, the *industry rivalry* in the form of substitute products as well as new market entrants, e.g., BigTechs such as Google, Amazon, and Apple, represents a relevant SF in the “co-creator of financial analysis,” “information aggregator,” “insourcer of sub-processes,” “lending community”, and “robo-advisory” business models. We can also observe this issue for “robo-advisory,” reflected in the business activities of FinTech Albert. Furthermore, in the specific case of “information aggregator,” negative *market conditions* factors such as market shocks influence the success of financial e-marketplaces since these external factors can affect the price of commodities, the trading volume, and the willingness of brokers to participate in digital markets (e.g., O’Reilly & Finnegan, 2010).

## Theoretical, methodological, and practical implications

**Fig. 4:**
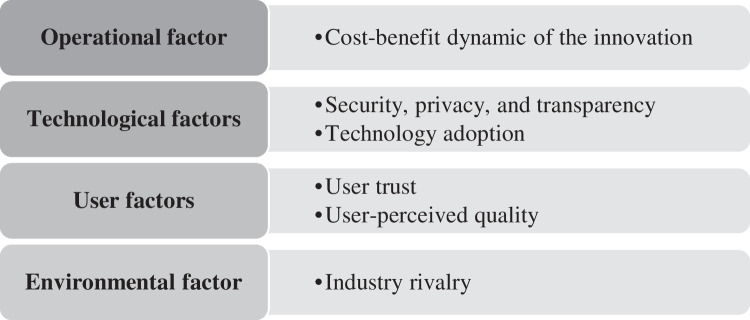
“Grand Challenges” within the FinTech ecosystem as identified by the taxonomy-based analysis

From a theoretical view, our taxonomy of FinTech SFs contributes to the existing knowledge of FinTech by synthesizing content from 231 academic publications about potentially relevant SFs for FinTechs. The study provides a conceptual structure and terminology to SFs for FinTechs in different archetypes in the form of a taxonomic structure. Compared to the existing literature on FinTechs, we contribute to the streams of FinTech and IS literature by providing a structured content-based analysis of industry-specific SFs for different business model archetypes provided by Eickhoff et al. (2017). We identified 31 factors associated with FinTech success derived from the interdisciplinary knowledge embedded within the extant scientific literature. While these 31 factors are diverse and show a wide spread of different SFs, we identified six “grand challenges” within the FinTech ecosystem, namely “cost–benefit dynamic of the innovation,” “technology adoption,” “security, privacy, and transparency,” “user trust,” “user-perceived quality,” and “industry rivalry” that has been studied and identified by the past researchers. These “grand challenges” within the ecosystem are visualized in Fig. 4 and are arranged with their associated dimension:

While FinTechs are “technology-driven” (e.g., Gomber et al., 2018), it is not surprising that our examination reveals technological factors as crucial for the success of six of the eight archetypes. However, this uniqueness of technological factors is not a necessary condition for success. As identified by our examination, e.g., within the archetype of “robo-advisory” and past taxonomic approaches in the FinTech sector (Drasch et al., 2018; Gimpel et al., 2018), success remains a composition and configuration of many factors that can potentially be relevant for FinTech survival. Our examination shows that while FinTech companies can take internal actions to respond to the “grand challenges” strategically, the impact of external effects such as *regulation* can counteract these efforts and influence the survival and growth of some FinTech business models in countries with restrictive legal and regulatory systems, such as within the European Union.

However, our study and the results derived can explain the differences and similarities between SFs as a first step, uncover knowledge gaps that have been overlooked and not studied so far (e.g., Gazel & Schwienbacher, 2020; Soto-Simeone et al. 2020). While our review shows that these factors were researched extensively, our study can serve as an ignition for more tailored research and examinations of other factors potentially relevant to the success of FinTechs. Besides the empirical derivation of SFs within the FinTech area, we illustrate the degree of generality of these characteristics and provide insights on their effect on diverse FinTech business model archetypes at seven distinct dimensions of success, thereby addressing a future research direction pointed out by Eickhoff et al. (2017). We validate these identified factors by comparing and discussing them in the context of successful real-world FinTech companies. In addition, we evaluated the usefulness of our findings with two individuals from the FinTech ecosystem. The participants that were interviewed confirmed the SFs identified as relevant and comprehensive.

From a methodological standpoint, we combine the taxonomic approach by Nickerson et al. (2013) with content analysis as a technique to identify SFs. In this manner, the taxonomic approach guided our systematic classification of SFs, and the content analysis methodically allowed us to identify relevant dimensions and characteristics. With this structured approach, we could review and abstract the findings of the extant scientific literature more effectively and provide an overview of how the identified SFs have been studied so far in the academic literature. We transfer our findings from the review process to real-world cases from the FinTech sector, showing the appropriateness of some SFs within a market context. Therefore, case-based taxonomy validation was useful to show how to transfer findings from past literature provided by taxonomies to real-world cases and practical settings.

From the practitioner’s perspective, potential founders and stakeholders of FinTechs can use our taxonomy to obtain a holistic overview of the relevant factors that contribute to the success of each FinTech business model archetype. Furthermore, interested practitioners can find and deviate a prioritization of possibly relevant SFs that maintain the survival of their own venture. Likewise, already-founded FinTechs can assess and refine their business activities and current capabilities based on our identified SFs to detect potential shortcomings in their business logic and value networks to achieve competitive business models. Additionally, our taxonomy can serve as a discussion platform and supplementary knowledge base for members of the financial ecosystem, e.g., banks, FinTech hubs, consultants, or venture capitalists, to identify the possibly relevant SFs across distinct business model archetypes in the FinTech ecosystem.

## Limitations and future research directions

Although our study is based on the widely accepted frameworks and procedures of Nickerson et al. (2013) and Webster and Watson (2002), the criteria applied during the literature review may impact the comprehensiveness of the results. This is especially the case for the business model archetypes of robo-advisors (*n* = 4*)* and information aggregators (*n* = 3). Results for these archetypes are based on a small sample of identified papers and a real-world example. Therefore, this study’s inferences should be treated carefully in light of this limitation. Among these criteria is the time restriction to include only scientific articles published from 2008 onward and the inclusion of only scientific articles published in leading peer-reviewed academic business research journals and conference proceedings. Additionally, potential limitations related to the taxonomic approach must be considered. For example, it is impossible to affirm completeness in the case of all relevant SFs of FinTech since we examined the literature within our inclusion criteria and stopped the empirical analysis after all adopted taxonomic objective and subjective ending conditions were met. Furthermore, the definition of meta-characteristic or ending conditions in taxonomy development frameworks is part of a subjective process based on value judgments made by the authors (Nickerson et al., 2013).

We encourage researchers interested in FinTech, especially in FinTech success, to build on our findings that leave room for future research. Although the original goal of a taxonomy is not to build theory or to identify, for example, causal relationships between its composing objects, the integrative knowledge resulting from this analysis can be used in future research to discern relationships between the identified SFs at different levels or be used to support theory building processes, also mentioned by Kundisch et al. (2021).

Further research can also build on our insights and enrich our taxonomy of FinTech SFs with causal explanations or testable propositions (Gregor, 2006; Muntermann et al., 2015) to assess the level of success criticality of the identified SFs either through different (i) FinTech lifecycle stages; (ii) archetype-specific product or service lifecycles; or (iii) in regards to diverse exploration–exploitation (corporate, productivity, competitive or marketing) strategies to gain a better understanding of the interconnection between strategic entrepreneurship and FinTech success. By providing a justified list of SFs and multi-dimensional conceptualization of FinTech success, this study also provides the basis for the development of strategic analysis tools, e.g., segment-based CPMs, to analyze the particular competitive position, strengths, and weaknesses of a FinTech company within the industry (e.g., David & David, 2017). We also validate our findings with (short) success stories with real-world examples from the FinTech sector to show important highlights from our results. Besides the archetypes “insourcer of sub-processes” and “robo-advisor,” we only focus on and discuss one SF. Consequentially, these cases are not complete assessments of the whole business model. A more detailed analysis of the configurations and SFs of FinTech business models in light of our results can be an avenue for future research. Our analysis is focused on FinTech SFs. Therefore, the cases contain FinTechs, that were able to show success with their business model and underlying activities. However, examining the success/failure dichotomy also constitutes an important supplementary line of research to be explored. While success and failure factors have no mutually opposing effects as a matter of principle (Dwivedi et al., 2015; Williams & Ramaprasad, 1996), the identification of relevant failure factors, their direct/indirect effects, and an investigation of failed FinTechs are important to avoid diverting into an incomplete strategic planning perspective, i.e., “complementarities trap” (Pettigrew et al., 2003).

Furthermore, in line with our research goal, we chose a conceptual-to-empirical approach as a starting point for our taxonomic process. However, a future alternative taxonomy can emerge from an empirical-to-conceptual approach to obtain a different perspective, e.g., by conducting expert interviews or focus groups with practitioners in the financial ecosystem. A comparison of both taxonomies contributes to identifying existent research-to-practice gaps. Also, because we choose one FinTech per archetype to reflect and validate our results from taxonomy, further research can choose a more comprehensive way of discussing real-world examples from the FinTech industry with the results presented.

## Conclusions

In this study, we provide insights into the potential determinants of FinTech success through a taxonomy-based analysis of 231 research articles built on empirically validated FinTech business model archetypes. Our analysis shows that technological characteristics such as “security, privacy, and transparency” and “technology adoption,” along with user factors such as “user trust” and “user-perceived quality” and operational factors as the “cost–benefit dynamic of the innovation” are relevant for FinTech success. At a more specific archetypal level, we identified (i) “security, privacy, and transparency” as the most relevant SF for the “cryptocurrency,” “co-creator of financial analysis,” “insourcer of sub-processes”, and “robo-advisor” archetypes; (ii) “technology adoption” as the most relevant SF for the FinTech business model archetypes of “alternative trading venue” and “lending community”; and (iii) the “user trust,” “user-perceived quality”, and “cost–benefit dynamic of the innovation” as the most relevant factors for the success of the FinTech business model archetypes “payment services,” “robo-advisor,” and “information aggregator,” respectively. Furthermore, we validate and discuss our identified factors with examples from the FinTech industry. Also, we evaluated the usefulness of our findings with two individuals from the FinTech ecosystem. Compared to previous literature, we contribute to the need within the streams of FinTech and IS literature by providing a structured content-based analysis of industry-specific SFs for FinTechs that can explain the differences and similarities between them and uncover knowledge gaps (e.g., Gazel & Schwienbacher, 2020; Soto-Simeone et al. 2020). As a result, the taxonomy can be used to solve practical problems, i.e., identifying potentially relevant SFs for FinTechs. The insights gained in this study suggest that the SFs play an important role in the success of FinTechs and should therefore be treated as a priority for the sustainable development of FinTech business models.

## Supplementary Information

Below is the link to the electronic supplementary material.

Supplementary file1 (DOCX 466 KB)

## Data Availability

(Anonymized) data and analysis that support the findings of this study are available on request by the corresponding author.
