# Supplementary material for: What determines FinTech success?—A taxonomy-based analysis of FinTech success factors
Source: Electron Mark. 2023 May 19;33(1):21. doi: 10.1007/s12525-023-00626-7 (PMC10197061; doi:10.1007/s12525-023-00626-7)
Supplement: Supplementary file 1 — Supplementary file1 (DOCX 466 KB) [file 12525_2023_626_MOESM1_ESM.docx]

******

**What determines FinTech success? -**

**A taxonomy-based analysis of FinTech success factors**

**[Online Appendix]**

This document contains the separate tables of the article "What determines FinTech success? - A taxonomy-based analysis of FinTech success factors", which has been submitted for review to the Electronic Markets (EM) Journal.

[Table A. Research Articles on FinTech Success Factors 2](#_Toc115798701)

[Table B. Search Keywords for each FinTech Business Model Archetype 12](#_Toc115798702)

[Table C. Taxonomy Development Process (Dimension and Characteristic Level) 13](#_Toc115798703)

[Table D. Ending Conditions met by each Iteration 15](#_Toc115798704)

Table A lists all relevant papers identified in our study, clustered by our used archetypes of FinTech business models. It presents the authors, the year of publication, the title of the publication, the publication venues, their VHB-JOURQUAL 3 (Henning-Thurau et al., 2023) ranking, the respective disciplines and the used research approach.

The final dataset of the scientific articles used for the taxonomy-based analysis (n= 231) contains relatively recent articles, with approximately 75% published after 2015. Nonetheless, despite the interdisciplinary nature of the FinTech phenomenon, scientific knowledge has grown in a segregated manner (Gomber et al., 2018).

Table A. Research Articles on FinTech Success Factors

| Author(s) |  | Year | Title | Venue  (Ranking) | Disci-pline | Research approach |
| --- | --- | --- | --- | --- | --- | --- |
|  | ***Alternative trading venue (87 Articles)*** | | | | | |
| **Ahlers et al.** |  | 2015 | Signaling in equity crowdfunding | Journal  (A) | TIE | Qualitative |
| **Albrecht et al.** |  | 2020 | The behavior of blockchain ventures on Twitter as a determinant for funding success | Journal  (B) | TIE | Qualitative |
| **Allison et al.** |  | 2015 | Crowdfunding in a prosocial microlending environment: examining the role of intrinsic versus extrinsic cues | Journal  (A) | TIE | Quantitative |
| **Allison et al.** |  | 2017 | Persuasion in crowdfunding. An elaboration likelihood model of crowdfunding performance | Journal  (A) | TIE | Qualitative |
| **Bade & Krezdorn** |  | 2018 | Cohesion among crowd investors in the presence of moral hazard | Journal  (C) | TIE | Quantitative |
| **Belleflamme et al.** |  | 2014 | Crowdfunding: Tapping the right crowd | Journal  (A) | TIE | Qualitative |
| **Block et al.** |  | 2018 | Which updates during an equity crowdfunding campaign increase crowd participation? | Journal  (B) | TIE | Quantitative |
| **Bogusz et al.** |  | 2019 | Designed entrepreneurial legitimacy: The case of a swedish crowdfunding platform | Journal  (A) | WI | Qualitative |
| **Bollaert et al.** |  | 2020 | The narcissism of crowdfunding entrepreneurs | Journal  (B) | TIE | Quantitative |
| **Bruton et al.** |  | 2015 | \| New financial alternatives in seeding entrepreneurship: microfinance, crowdfunding, and peer-to-peer innovations \| \| --- \| | Journal  (A) | TIE | Quantitative |
| **Burtch et al.** |  | 2013 | An empirical examination of the antecedents and consequences of contribution patterns in crowd-funded markets | Journal  (A+) | WI | Quantitative |
| **Burtch et al.** |  | 2016 | Secret admirers: an empirical examination of information hiding and contribution dynamics in online crowdfunding | Journal  (A+) | WI | Quantitative |
| **Butticè et al.** |  | 2017 | Serial crowdfunding, social capital, and project success | Journal  (A) | TIE | Qualitative |
| **Cai et al.** |  | 2016 | The price of street friends: social networks, informed trading, and shareholder costs | Journal  (A) | BA-FI | Quantitative |
| **Chan & Parhankangas** |  | 2017 | Crowdfunding innovative ideas: How incremental and radical innovativeness influence funding outcomes | Journal  (A) | TIE | Mixed Methods |
| **Chan et al.** |  | 2018 | Reward-based crowdfunding success: Decomposition of the project, product category, entrepreneur, and location effects | Journal  (C) | TIE | Qualitative |
| **Cholokova & Clarysse** |  | 2015 | Does the possibility to make equity investments in crowdfunding projects crowd out reward-based investments? | Journal  (A) | TIE | Quantitative |
| **Clauss et al.** |  | 2020 | Increasing crowdfunding success through social media: The importance of reach and utilisation in reward-based crowdfunding | Journal  (B) | TIE | Quantitative |
| **Colombo et al.** |  | 2015 | Internal social capital and the attraction of early contributions in crowdfunding | Journal  (A) | TIE | Quantitative |
| **Courtney et al.** |  | 2017 | Resolving information asymmetry: Signaling, endorsement, and crowdfunding success | Journal  (A) | TIE | Quantitative |
| **Crosetto & Regner** |  | 2018 | It's never too late: Funding dynamics and self pledges in reward-based crowdfunding | Journal  (A) | TIE | Qualitative |
| **Culkin et al.** |  | 2016 | Critical innovations in the UK peer-to-peer (P2P) and equity alternative finance markets for small firm growth | Journal  (C) | TIE | Quantitative |
| **Cumming et al.** |  | 2021 | Does equity crowdfunding democratize entrepreneurial finance? | Journal  (B) | TIE | Quantitative |
| **Cummings et al.** |  | 2020 | An equity crowdfunding research agenda: Evidence from stakeholder participation in the rulemaking process | Journal  (B) | TIE | Quantitative |
| **Davis et al.** |  | 2017 | Funders' positive affective reactions to entrepreneurs' crowdfunding pitches: The influence of perceived product creativity and entrepreneurial passion | Journal  (A) | TIE | Quantitative |
| **Du et al.** |  | 2019 | The more options, the better? Investigating the impact of the number of options on backers’ decisions in reward-based crowdfunding projects | Journal  (C) | WI | Quantitative |
| **Du et al.** |  | 2019 | Promoting crowdfunding with lottery: the impact on campaign performance | Journal  (B) | WI | Quantitative |
| **Efrat & Gilboa** |  | 2020 | Relationship approach to crowdfunding: How creators and supporters interaction enhances projects’ success | Journal  (B) | WI | Quantitative |
| **Feola et al.** |  | 2021 | Segmenting “digital investors”: Evidence from the Italian equity crowdfunding market | Journal  (B) | TIE | Qualitative |
| **Fisch et al.** |  | 2021 | Motives and profiles of ICO investors | Journal  (B) | SM | Quantitative |
| **Gerber & Hui** |  | 2013 | Crowdfunding: Motivations and deterrents for participation | Journal  (B) | WI | Mixed Methods |
| **Giudici et al.** |  | 2018 | Reward-based crowdfunding of entrepreneurial projects: the effect of local altruism and localized social capital on proponents’ success | Journal  (B) | TIE | Quantitative |
| **Gleasure & Feller** |  | 2016 | Does Heart of Head Rule Donor Behaviors in Charitable Crowdfunding Markets? | Journal  (B) | WI | Qualitative |
| **Gleasure et al.** |  | 2017 | Inclusive technologies, selective traditions: a socio-material case study of crowdfunded book publishing | Journal  (A) | WI | Quantitative |
| **Gobble** |  | 2012 | Everyone is a venture capitalist: The new age of crowdfunding | Journal  (C) | TIE | Quantitative |
| **Haas & Blohm** |  | 2017 | Blueprinting crowdfunding: Designing a crowdfunding service configuration framework | Conference  (C) | WI | Qualitative |
| **Haddad & Hornuf** |  | 2019 | The emergence of the global Fintech market: Economic and technological determinants | Journal  (B) | TIE | Quantitative |
| **Hashim et al.** |  | 2017 | Information feedback, targeting, and coordination: an experimental study | Journal  (A+) | WI | Quantitative |
| **Hoegen et al.** |  | 2018 | How do investors decide? An interdisciplinary review of decision-making in crowdfunding | Journal  (B) | WI | Mixed Methods |
| **Hornuf & Neuenkirch** |  | 2017 | Pricing shares in equity crowdfunding | Journal  (B) | TIE | Quantitative |
| **Hornuf & Schwienbacher** |  | 2017 | Should securities regulation promote equity crowdfunding? | Journal  (B) | TIE | Quantitative |
| **Huang et al.** |  | 2020 | The geography of initial coin offerings | Journal  (B) | TIE | Mixed Methods |
| **Josefy et al.** |  | 2017 | The role of community in crowdfunding success: Evidence on cultural attributes in funding campaigns to "save the local theater" | Journal  (A) | TIE | Quantitative |
| **Kang et al.** |  | 2017 | Remarkable advocates: An investigation of geographic distance and social capital for crowdfunding | Journal  (B) | WI | Quantitative |
| **Kher et al.** |  | 2021 | Blockchain, Bitcoin, and ICOs: A review and research agenda | Journal  (B) | SM | Quantitative |
| **Kleinert et al.** |  | 2020 | Third-party signals in equity crowdfunding: the role of prior financing | Journal  (B) | SM | Mixed Methods |
| **Koch & Siering** |  | 2015 | Crowdfunding success factors: The characteristics of successfully funded projects on crowdfunding platforms | Conference  (B) | WI | Quantitative |
| **Koch et al.** |  | 2019 | The recipe of successful crowdfunding campaigns | Journal  (B) | WI | Qualitative |
| **Kromidha & Robson** |  | 2016 | Social identity and signaling success factors in online crowdfunding | Journal  (B) | TIE | Quantitative |
| **Kunz et al.** |  | 2017 | An empirical investigation of signaling in reward-based crowdfunding | Journal  (C) | WI | Quantitative |
| **Kuppuswamy & Bayus** |  | 2017 | Does my contribution to your crowdfunding project matter? | Journal  (A) | TIE | Quantitative |
| **Lee & Bian** |  | 2018 | Factors affecting success of serial crowdfunding: From heuristic and systematic perspectives | Conference  (C) | WI | Mixed Methods |
| **Lehner** |  | 2014 | The formation and interplay of social capital in crowdfunded social ventures | Journal  (B) | TIE | Mixed Methods |
| **Li et al.** |  | 2020 | A social recommendation approach for reward-based crowdfunding campaigns | Journal  (B) | WI | Qualitative |
| **Löher et al.** |  | 2018 | A research note on entrepreneurs’ financial commitment and crowdfunding success | Journal  (C) | TIE | Quantitative |
| **Lukkarinen et al.** |  | 2016 | Success drivers of online equity crowdfunding campaigns | Journal  (B) | WI | Quantitative |
| **Mamonov & Malaga** |  | 2018 | Success Factors in Title III Equity Crowdfunding in the United States | Journal  (B) | WI | Quantitative |
| **Masiak et al.** |  | 2020 | Initial coin offerings (ICOs): market cycles and relationship with bitcoin and ether | Journal  (B) | SM | Mixed Methods |
| **Miglo and Miglo** |  | 2019 | Market imperfections and crowdfunding | Journal  (B) | TIE | Mixed Methods |
| **Mitra & Euchner** |  | 2016 | Business acceleration at scale | Journal  (C) | TIE | Quantitative |
| **Mohammad & Shafi** |  | 2018 | Gender differences in the contribution patterns of equity-crowdfunding investors | Journal  (B) | TIE | Mixed Methods |
| **Mollick** |  | 2014 | The dynamics of crowdfunding: An exploratory study | Journal  (A) | TIE | Quantitative |
| **Momtaz** |  | 2021a | Entrepreneurial finance and moral hazard: Evidence from token offerings | Journal  (A) | WI | Qualitative |
| **Momtaz** |  | 2021b | Initial coin offerings, asymmetric information, and loyal CEOs | Journal  (B) | SM | Quantitative |
| **Parhankangas & Renko** |  | 2017 | Linguistic style and crowdfunding success among social and commercial entrepreneurs | Journal  (A) | TIE | Quantitative |
| **Piva & Rossi-Lamastra** |  | 2018 | Human capital signals and entrepreneurs' success in equity crowdfunding | Journal  (B) | TIE | Quantitative |
| **Polzin et al.** |  | 2018 | The wisdom of the crowd in funding: Information heterogeneity and social networks of crowdfunders | Journal  (B) | TIE | Qualitative |
| **Ralcheva & Roosenboom** |  | 2020 | Forecasting success in equity crowdfunding | Journal  (B) | TIE | Quantitative |
| **Roma et al.** |  | 2017 | From the crowd to the market: The role of reward-based crowdfunding performance in attracting professional investors | Journal  (A) | TIE | Quantitative |
| **Rose et al.** |  | 2021 | Launching for success: The effects of psychological distance and mental simulation on funding decisions and crowdfunding performance | Journal  (A) | TIE | Quantitative |
| **Ryoba et al.** |  | 2021 | Feature subset selection for predicting the success of crowdfunding project campaigns | Journal  (B) | WI | Quantitative |
| **Schückes & Gutmann** |  | 2021 | Why do startups pursue initial coin offerings (ICOs)? The role of economic drivers and social identity on funding choice | Journal  (B) | SM | Qualitative |
| **Shafi** |  | 2021 | Investors’ evaluation criteria in equity crowdfunding | Journal  (B) | TIE | Quantitative |
| **Skirnevskiy et al.** |  | 2017 | The influence of internal social capital on serial creator's success in crowdfunding | Journal  (A) | TIE | Qualitative |
| **Stanko & Henard** |  | 2017 | Toward a better understanding of crowdfunding, openness and the consequences for innovation | Journal  (A) | TIE | Quantitative |
| **Steigenberger** |  | 2017 | Why supporters contribute to reward-based crowdfunding | Journal  (C) | TIE | Qualitative |
| **Vismara** |  | 2016 | Equity retention and social network theory in equity crowdfunding | Journal  (B) | TIE | Quantitative |
| **Vismara** |  | 2018 | Information cascades among investors in equity crowdfunding | Journal  (A) | TIE | Quantitative |
| **Wang et al.** |  | 2018 | Understanding the importance of interaction between creators and backers in crowdfunding success | Journal  (C) | WI | Quantitative |
| **Wessel et al.** |  | 2016 | The emergence and effects of fake social information: Evidence from crowdfunding | Journal  (B) | WI | Quantitative |
| **Wessel et al.** |  | 2017 | Opening the floodgates: The implications of increasing platform openness in crowdfunding | Journal  (A) | WI | Quantitative |
| **Xie et al.** |  | 2019 | Success factors and complex dynamics of crowdfunding: An empirical research on Taobao platform in China | Journal  (B) | WI | Quantitative |
| **Yu et al.** |  | 2017 | Crowdfunding and regional entrepreneurial investment: An application of the Crowd Berkeley database | Journal  (A) | TIE | Quantitative |
| **Yuan et al.** |  | 2016 | The determinants of crowdfunding success: A semantic text analytics approach | Journal  (B) | WI | Quantitative |
| **Zheng et al.** |  | 2014 | The role of multidimensional social capital in crowdfunding: A comparative study in China and US | Journal  (B) | WI | Quantitative |
| **Zheng et al.** |  | 2017 | Project implementation success in reward-based crowdfunding: An empirical study | Journal  (B) | WI | Quantitative |
| **Zhou et al.** |  | 2018 | Project description and crowdfunding success: An exploratory study | Journal  (B) | WI | Quantitative |
|  | ***Co-creator of financial analysis (7 Articles)*** | | | | | |
| **Huang et al.** |  | 2020 | Initial evidence on the impact of big data implementation on firm performance | Journal  (B) | WI | Quantitative |
| **Liu et al.** |  | 2018 | The challenges of business analytics: Successes and failures | Conference  (C) | WI | Qualitative |
| **Mikalef et al.** |  | 2018 | A stage model for uncovering inertia in big data  analytics adoption | Conference  (C) | WI | Qualitative |
| **Miklaef et al.** |  | 2018 | Big data analytics capabilities: A systematic literature review and research agenda | Journal  (C) | WI | Qualitative |
| **Richins et al.** |  | 2017 | Big data analytics: Opportunity or threat for the accounting profession? | Journal  (C) | WI | Qualitative |
| **Watson** |  | 2018 | Revisiting Ralph Sprague’s framework for developing decision support systems | Journal  (C) | WI | Qualitative |
| **Wiener et al.** |  | 2020 | Big-data business models: A critical literature review and multiperspective research framework | Journal  (A) | WI | Qualitative |
|  | ***Cryptocurrency (15 Articles)*** | | | | | |
| **Cousins et al.** |  | 2019 | A value-sensitive design perspective of cryptocurrencies: a research agenda | Journal  (C) | WI | Qualitative |
| **Cusumano** |  | 2014 | Technology strategy and management. The Bitcoin ecosystem. Speculating on how the bitcoin economy might evolve. | Journal  (B) | WI | Quantitative |
| **Eyal & Sirer** |  | 2018 | Majority is not enough: Bitcoin mining is vulnerable | Journal  (B) | WI | Qualitative |
| **Genkin et al.** |  | 2018 | Privacy in decentralized cryptocurrencies | Journal  (B) | WI | Qualitative |
| **Guo et al-** |  | 2011 | Virtual wealth protection through virtual money exchange | Journal  (C) | WI | Qualitative |
| **Kugerr** |  | 2018 | Why cryptocurrencies use so much energy—and what to do about it | Journal  (B) | WI | Qualitative |
| **Li & Wang** |  | 2017 | The technology and economic determinants of cryptocurrency exchange rates: The case of Bitcoin | Journal  (B) | WI | Mixed Methods |
| **Mai et al.** |  | 2018 | How does social media impact Bitcoin value? A test of the silent majority hypothesis | Journal  (A) | WI | Quantitative |
| **Meiklejohn et al.** |  | 2013 | A fistful of Bitcoins: Characterizing payments among men with no names | Journal  (B) | WI | Quantitative |
| **Mendoza‑Tello et al.** |  | 2019 | Disruptive innovation of cryptocurrencies in consumer acceptance and trust | Journal  (C) | WI | Quantitative |
| **Narayanan & Clark** |  | 2017 | Bitcoin’s academic pedigree | Journal  (B) | WI | Quantitative |
| **Underwood** |  | 2016 | Blockchain beyond bitcoin | Journal  (B) | WI | Mixed Methods |
| **van Alstyne** |  | 2014 | Economic and business dimensions why bitcoin has value: Evaluating the evolving controversial digital currency | Journal  (B) | WI | Qualitative |
| **Weaver** |  | 2018 | Inside risks of cryptocurrencies | Journal  (B) | WI | Quantitative |
| **Zohar** |  | 2015 | Bitcoin under the hood | Journal  (B) | WI | Qualitative |
|  | ***Information aggregator (3 Articles)*** | | | | | |
| **Aswani et al.** |  | 2018 | Search engine marketing is not all gold: Insights from Twitter and SEO Clerks | Journal  (C) | WI | Mixed-Methods |
| **O’Reilly & Finnegan** |  | 2010 | Intermediaries in inter-organizational networks: building a theory of electronic marketplace performance | Journal  (A) | WI | Qualitative |
| **Thitimajshim et al.** |  | 2018 | A framework to identify factors affecting the performance of third-party B2B e-marketplaces: A seller’s perspective | Journal  (B) | WI | Quantitative |
|  | ***Insourcer of sub-processes (20 Articles)*** | | | | | |
| **Alzadjali & Elbanna** |  | 2020 | Smart institutional intervention in the adoption of digital infrastructure: The case of government cloud computing in Oman | Journal  (B) | WI | Mixed-Methods |
| **Bouaynaya** |  | 2020 | Characterization of cloud computing reversibility as explored by the Delphi method | Journal  (B) | WI | Mixed-Methods |
| **Brandt et al.** |  | 2012 | Autonomic management of software as a service systems with multiple quality of service classes | Conference  (B) | WI | Quantitative |
| **Buyya et al.** |  | 2019 | A manifesto for future generation cloud computing: research directions for the next decade | Journal  (A) | WI | Mixed-Methods |
| **Chou & Chiang** |  | 2013 | Understanding the formation of software-as-a-service (SaaS) satisfaction from the perspective of service quality | Journal  (B) | WI | Quantitative |
| **Dizdarević et al.** |  | 2019 | A survey of communication protocols for internet  of things and related challenges of fog and cloud  computing integration | Journal  (A) | WI | Mixed-Methods |
| **El-Haddadeh** |  | 2020 | Digital innovation dynamics influence on organisational adoption: the case of cloud computing services | Journal  (B) | WI | Qualitative |
| **Ferri et al.** |  | 2020 | Cloud computing in high tech startups: evidence from a case study | Journal  (C) | SM | Quantitative |
| **Garrison et al.** |  | 2012 | Success factors for deploying cloud computing | Journal  (B) | WI | Mixed-Methods |
| **Garrison et al.** |  | 2015 | The effects of IT capabilities and delivery model on cloud computing success and firm performance for cloud supported processes and operations | Journal  (C) | WI | Qualitative |
| **Gill & Buyya** |  | 2019 | A taxonomy and future directions for sustainable cloud computing: 360 degree view | Journal  (A) | WI | Qualitative |
| **Kauffman et al.** |  | 2018 | A metrics suite of cloud computing adoption readiness | Journal  (B) | WI | Qualitative |
| **Kumar & Kumar** |  | 2019 | Issues and challenges of load balancing techniques  In cloud computing: A survey | Journal  (A) | WI | Quantitative |
| **Lang & Wiesche** |  | 2018 | Criteria for selecting cloud service providers: A Delphi study of quality-of- service attributes | Journal  (B) | WI | Qualitative |
| **Lee et al.** |  | 2013 | Using balanced scorecards for the evaluation of "Software-as-a-service" | Journal  (B) | WI | Qualitative |
| **Loukis et al.** |  | 2019 | Determinants of software-as-a-service benefits and impact on firm performance | Journal  (B) | WI | Quantitative |
| **Rieger et al.** |  | 2013 | Cloud-Computing in Banking: Influential factors, benefits and risks from a decision maker’s perspective | Conference  (C) | WI | Qualitative |
| **Trenz et al.** |  | 2013 | The role of uncertainty in cloud computing continuance: antecedents, mitigators, and consequences | Conference  (B) | WI | Quantitative |
| **Walther et al.** |  | 2018 | Should we stay or should we go? Analyzing continuance of cloud enterprise systems | Journal  (C) | WI | Qualitative |
| **Zhou & Buyya** |  | 2019 | Augmentation techniques for mobile cloud computing: A taxonomy, survey, and future directions | Journal  (A) | WI | Mixed-Methods |
|  | ***Lending community (19 Articles)*** | | | | | |
| **Arnold et al.** |  | 2021 | Refinancing MFIs with market power: theory and evidence | Journal  (B) | SM | Mixed-Methods |
| **Assadi & Hudson** |  | 2010 | Marketing-mix of online social lending websites | Journal  (C) | WI | Quantitative |
| **Burtch et al.** |  | 2014 | Cultural differences and geography as determinants of online prosocial lending | Journal  (A+) | WI | Quantitative |
| **Chen et al.** |  | 2014 | A trust model for online peer-to-peer lending: A lender’s perspective | Journal  (C) | WI | Mixed-Methods |
| **Chen et al.** |  | 2016 | Are investors rational or perceptual in P2P lending? | Journal  (C) | WI | Qualitative |
| **Chen et al.** |  | 2017 | Gender discrimination in online peer-to-peer credit lending: evidence from a lending platform in China | Journal  (C) | WI | Quantitative |
| **Chen et al.** |  | 2021 | Borrower learning effects: Do prior experiences promote continuous successes in peer-to-peer lending? | Journal (B) | WI | Quantitative |
| **Eldridge et al.** |  | 2021 | What impact does equity crowdfunding have on SME innovation and growth? An empirical study | Journal  (B) | SM | Mixed-Methods |
| **Feller et al.** |  | 2017 | Information sharing and user behavior in internet-enabled peer-to-peer lending systems: An empirical study | Journal  (A) | WI | Quantitative |
| **Gao et al.** |  | 2018 | The performance of the P2P finance industry in China | Journal  (B) | WI | Quantitative |
| **Jagtiani et al.** |  | 2018 | Do Fintech lenders penetrate areas that are underserved by traditional banks | Journal  (C) | SM | Mixed-Methods |
| **Johnson et al.** |  | 2010 | Online or offline? The rise of “peer-to-peer” lending in microfinance | Journal  (C) | WI | Quantitative |
| **Kgoroeadira et al.** |  | 2019 | Small business online loan crowdfunding: who gets funded and what determines the rate of interest | Journal  (B) | SM | Quantitative |
| **Liu et al.** |  | 2015 | Friendship in online peer-to-peer lending: Pipes, prisms, and relational herding | Journal  (A+) | WI | Quantitative |
| **Ma et al.** |  | 2018 | A new aspect on P2P online lending default prediction using meta-level phone usage data in China | Journal  (B) | WI | Quantitative |
| **Serrano-Cinca & Gutiérrez-Nieto** |  | 2016 | The use of profit scoring as an alternative to credit scoring systems in peer-to-peer (P2P) lending | Journal  (B) | WI | Qualitative |
| **Tao et al.** |  | 2017 | Who can get money? Evidence from the Chinese peer-to-peer lending platform | Journal  (B) | WI | Quantitative |
| **Wang et al.** |  | 2018 | A novel behavioral scoring model for estimating probability of default over time in peer-to-peer lending | Journal  (B) | WI | Quantitative |
| **Ye et al.** |  | 2018 | Loan evaluation in P2P lending based on Random Forest optimized by genetic algorithm with profit score | Journal  (B) | WI | Quantitative |
|  | ***Payment service – Billing (1 Article)*** | | | | | |
| **Cuylen et.al.** |  | 2016 | Development of a maturity model for electronic invoice processes | Journal  (B) | WI | Mixed-Methods |
|  | ***Payment service – Mobile (52 Articles)*** | | | | | |
| **Albashrawi and Motiwalla** |  | 2019 | Privacy and personalization in continued usage intention of mobile banking: An integrative perspective | Journal  (B) | WI | Qualitative |
| **Apanasevic** |  | 2013 | Factors influencing the slow rate of penetration of NFC mobile payment in Western Europe | Conference  (B) | WI | Qualitative |
| **Au & Kauffman** |  | 2008 | The economics of mobile payments: Understanding stakeholder issues for an emerging financial technology application | Journal  (C) | WI | Mixed-Methods |
| **Balocco et al.** |  | 2008 | Mobile payment applications: An exploratory analysis of the Italian diffusion process. | Conference  (B) | WI | Qualitative |
| **Bucherer et al.** |  | 2012 | Towards systematic business model innovation: Lessons from product innovation management | Journal  (C) | TIE | Qualitative |
| **Chun et al.** |  | 2016 | Transaction security investments in online marketplaces: An analytical examination of financial liabilities | Journal  (B) | WI | Quantitative |
| **Dahlberg et al.** |  | 2015 | M-payment- How disruptive technologies could change the payment ecosystem | Conference  (B) | WI | Quantitative |
| **David-West et al.** |  | 2018 | A resource-based view of digital ﬁnancial services (DFS): An exploratory study of Nigerian providers | Journal  (B) | SM | Qualitative |
| **Dercks et al.** |  | 2018 | From chaining blocks to breaking even: A study on the profitability of Bitcoin mining from 2012 to 2016 | Journal  (B) | WI | Quantitative |
| **De Reuver et al.** |  | 2015 | Stakeholder preferences for mobile payment security platforms- understanding trade-offs between SIM, embedded and cloud-based secure elements | Conference  (B) | WI | Mixed-Methods |
| **Du** |  | 2018 | Complacency, capabilities, and institutional pressure: Understanding financial institutions’ participation in the nascent mobile payments ecosystem | Journal  (B) | WI | Mixed-Methods |
| **Foster & Heeks** |  | 2013 | Innovation and scaling of ICT for the bottom-of-the-pyramid | Journal  (A) | WI | Mixed-Methods |
| **Hedman & Henningsson** |  | 2012 | Competition and collaboration shaping the digital payment infrastructure | Conference  (C) | WI | Qualitative |
| **Hillman et al.** |  | 2014 | User challenges and successes with mobile payment services in North America | Journal  (C) | WI | Quantitative |
| **Iman** |  | 2018 | Is mobile payment still relevant in the Fintech era? | Journal  (C) | WI | Quantitative |
| **Islam et al.** |  | 2018 | Does mobile money use increase firms’ investment? Evidence from enterprise surveys in Kenya, Uganda, and Tanzania | Journal  (B) | SM | Quantitative |
| **Jaspers et al.** |  | 2012 | Organizing interindustry architectural innovations: Evidence from mobile communication applications | Journal  (A) | TIE | Mixed-Methods |
| **Karjalouto et al.** |  | 2019 | How perceived value drives the use of mobile financial services apps | Journal  (C) | WI | Qualitative |
| **Kazan** |  | 2015 | The innovative capabilities of digital payment platforms: A comparative study of Apple Pay & Google Wallet | Conference  (B) | WI | Qualitative |
| **Kazan & Damsgaard** |  | 2014 | An investigation of digital payment platform designs: A comparative study of four European solutions | Conference  (B) | WI | Qualitative |
| **Kazan et al.** |  | 2015 | Disentangling competition among platform driven strategic groups- A comparative case study of UK mobile payment platforms | Conference  (B) | WI | Qualitative |
| **Lashitew et al.** |  | 2019 | Mobile phones for financial inclusion: What explains the diffusion of mobile money innovations? | Journal  (A) | TIE | Quantitative |
| **Liébana-Cabanillas et al.** |  | 2015 | Behavioral model of younger users in m-payment systems | Journal  (C) | WI | Qualitative |
| **Lin et al.** |  | 2014 | Understanding the evolution of consumer trust in mobile commerce: A longitudinal study | Journal (C) | WI | Qualitative |
| **Liu et al.** |  | 2015 | Competition, cooperation, and regulation: Understanding the evolution of the mobile payments technology ecosystem | Journal  (C) | WI | Qualitative |
| **Liu et al.** |  | 2019 | The impact of mobility, risk, and cost on the users’ intention to adopt mobile payments | Journal  (C) | WI | Qualitative |
| **Lu et al.** |  | 2011 | Dynamics between the trust transfer process and intention to use mobile payment services: A cross-environment perspective | Journal  (B) | WI | Qualitative |
| **Markendahl** |  | 2013 | Change of market structure for mobile payments services in Sweden- the case of SMS tickets | Conference  (B) | WI | Qualitative |
| **Moroni et al.** |  | 2015 | Adoption factors of NFC mobile proximity payments in Italy | Conference  (C) | WI | Qualitative |
| **Mou et al.** |  | 2017 | Trust and risk in consumer acceptance of e-services | Journal  (C) | WI | Qualitative |
| **O’Reilly et al.** |  | 2012 | To M-Pay or not to M-Pay - Realising the potential of smart phones: Conceptual modeling and empirical validation | Journal  (B) | WI | Qualitative |
| **Ondrus** |  | 2015 | Clashing over the NFC secure element for platform leadership in the mobile payment ecosystem | Conference  (C) | WI | Qualitative |
| **Ondrus & Lyytinen** |  | 2011 | Mobile payments market: Towards another clash of the titans? | Conference  (B) | WI | Qualitative |
| **Ondrus & Pigneur** |  | 2009 | Near field communication: An assessment for future payment systems | Journal  (C) | WI | Qualitative |
| **Ondrus et al.** |  | 2009 | Why mobile payments fail? Towards a dynamic and multi-perspective explanation | Conference  (C) | WI | Qualitative |
| **Ondrus et al.** |  | 2015 | The impact of openness on the market potential of multi-sided platforms: A case study of mobile payment platforms | Journal  (A) | WI | Qualitative |
| **Ozcan & Santos** |  | 2015 | The market that never was: Turf wars and failed alliances in mobile payments | Journal  (A) | SM | Qualitative |
| **Park & Islam** |  | 2014 | The potential and limitations of a public mobile payment service: Did Bangladesh electronic money transfer system make a difference in “unbanked” communities? | Conference  (C) | WI | Qualitative |
| **Pastoll et al.** |  | 2014 | Starbucks Canada: The mobile payments decision | Conference  (A) | WI | Mixed-Methods |
| **Pousttchi et al.** |  | 2009 | Proposing a comprehensive framework for analysis and engineering of mobile payment business models | Journal  (C) | WI | Mixed-Methods |
| **Puschmann** |  | 2017 | Fintech | Journal  (B) | WI | Quantitative |
| **Rukanova et al.** |  | 2020 | Emergence of collective digital innovations through the process of control point driven network reconfiguration and reframing: the case of mobile payment | Journal  (B) | WI | Mixed-Methods |
| **Shao et al.** |  | 2019 | Antecedents of trust and continuance intention in mobile payment platforms: The moderating effect of gender | Journal  (C) | WI | Quantitative |
| **Shin** |  | 2010 | Modeling the interaction of users and mobile payment system: conceptual framework. | Journal  (C) | WI | Quantitative |
| **Singh & Sinha** |  | 2020 | How perceived trust mediates merchant's intention to use a mobile wallet technology | Journal  (C) | SM | Quantitative |
| **Staykova & Damsgaard** |  | 2014 | A model of digital payment infrastructure formation and development- the EU regulator´s perspective | Conference  (B) | WI | Mixed-Methods |
| **Staykova & Damsgaard** |  | 2015 | Introducing reach and range for digital payment platforms | Conference  (B) | WI | Quantitative |
| **Trütsch** |  | 2016 | The impact of mobile payment on payment choice | Journal  (C) | BA-FI | Mixed-Methods |
| **Wakamori & Welte** |  | 2017 | Why do shoppers use cash? evidence from shopping diary data | Journal  (A) | BA-FI | Qualitative |
| **Wooder & Baker** |  | 2012 | Extracting key lessons in service innovation | Journal  (A) | TIE | Mixed-Methods |
| **Zarmpou et al.** |  | 2012 | Modeling users’ acceptance of mobile services | Journal  (C) | WI | Quantitative |
| **Zhou** |  | 2013 | An empirical examination of continuance intention of mobile payment services | Journal  (B) | WI | Quantitative |
|  | ***Payment service – Online (23 Articles)*** | | | | | |
| **Barkhordari et al.** |  | 2017 | Factors influencing adoption of e-payment systems: An empirical study on Iranian customers | Journal  (C) | WI | Mixed-Methods |
| **Blaschke et al.** |  | 2019 | Design principles for digital value co-creation networks: a service-dominant logic perspective | Journal  (B) | WI | Qualitative |
| **Bons et al.** |  | 2012 | Banking in the Internet and mobile era | Journal  (B) | WI | Qualitative |
| **Bucherer et al.** |  | 2012 | Towards systematic business model innovation: Lessons from product innovation management | Journal  (C) | TIE | Qualitative |
| **Dai & Salam** |  | 2014 | Does service convenience matter? An empirical assessment of service quality, service convenience and exchange relationship in electronic mediated environment | Journal  (B) | WI | Mixed-Methods |
| **Gozman et al.** |  | 2018 | The innovation mechanisms of Fintech start-ups: insights from SWIFT´s Innotribe competition | Journal  (A) | WI | Quantitative |
| **Hedman & Henningsson** |  | 2012 | Competition and collaboration shaping the digital payment infrastructure | Conference  (C) | WI | Mixed-Methods |
| **Hjelholt & Damsgaard** |  | 2012 | The genesis and evolution of digital payment platforms | Conference  (B) | WI | Qualitative |
| **Hong & Cha** |  | 2013 | The mediating role of consumer trust in an online merchant in predicting purchase intention | Journal  (C) | WI | Qualitative |
| **Kazan & Damsgaard** |  | 2014 | An Investigation of Digital Payment Platforms Designs: A Comparative study Of Four European Solutions | Conference  (C) | WI | Quantitative |
| **Kazan & Damsgaard** |  | 2016 | Towards a market entry framework of digital payment platforms | Journal  (C) | WI | Mixed-Methods |
| **Kim et al.** |  | 2010 | An empirical study of customers’ perceptions of security and trust in e-payment systems | Journal  (C) | WI | Mixed-Methods |
| **Kim et al.** |  | 2016 | Web assurance seal services, trust and consumers´ concerns: An investigation of e-commerce transaction intentions across two nations | Journal  (A) | WI | Quantitative |
| **Liang et al.** |  | 2017 | The nature of sellers´ cyber credit in C2C e-commerce: The perspective of social capital | Journal  (C) | WI | Quantitative |
| **Mou et al.** |  | 2017 | Trust and risk in consumer acceptance of e-services | Journal  (C) | WI | Quantitative |
| **Ogbanufe & Kim** |  | 2018 | Comparing fingerprint-based biometrics authentication versus traditional authentication methods for e-payment | Journal  (B) | WI | Quantitative |
| **Ozcan & Santos** |  | 2015 | The market that never was: Turf wars and failed alliances in mobile payments | Journal  (A) | SM | Quantitative |
| **Rimba et al.** |  | 2020 | Quantifying the cost of distrust: Comparing Blockchain and Cloud services for business process execution | Journal  (B) | WI | Quantitative |
| **Scott et al.** |  | 2017 | The long-term effect of digital innovation on bank performance: an empirical study of SWIFT adoption in financial services | Journal  (A) | TIE | Quantitative |
| **Treiblmaier et al.** |  | 2008 | Success factors of internet payment systems | Journal  (C) | WI | Quantitative |
| **Xu & Riedl** |  | 2011 | Understanding online payment method choice: An eye-tracking study | Conference  (A) | WI | Quantitative |
| **Yuan et al.** |  | 2019 | Continuous usage intention of internet banking: A commitment‑trust model | Journal  (B) | WI | Quantitative |
| **Zhang et al.^[[1]](#footnote-2)^** |  | 2020 | The construction and simulation of internet financial product diffusion model based on complex network and consumer decision‑making mechanism | Journal  (B) | WI | Quantitative |
|  | ***Robo-advisor (4 Articles)*** | | | | | |
| **Beketov et al.** |  | 2018 | Robo Advisors: Quantitative methods inside the robots | Journal  (B) | BA-FI | Quantitative |
| **Bruckes et al.** |  | 2019 | Determinants and barriers of adopting robo-advisory services | Conference  (A) | WI | Quantitative |
| **Jung et al.** |  | 2018 | Designing a robo-advisor for risk-averse, low-budget consumers | Journal  (B) | WI | Qualitative |
| **Tauchert & Mesbah** |  | 2019 | Following the robot? Investigating users’ utilization of advice from robo-advisors | Conference  (A) | WI | Quantitative |

Table B. Search Keywords for each FinTech Business Model Archetype

| Archetype | Keywords |
| --- | --- |
| Alternative trading venue | *Crowdfunding:* "crowdfunding" OR "donation based crowdfunding" OR "initial coin offering" OR "security token offering"  *Online credit:* "online credit" OR "reward based crowdfunding"  *Trading community:* "trading community" OR "investment community" OR "stock community" |
| Co-creator of financial analysis | "financial analysis app" OR "predictive analytics app" OR "financial analytics" OR "predictive analytics" OR "data analytics" |
| Cryptocurrency | "digital money" OR "digital currency" OR "virtual currency" OR "cryptocurrency" OR "electronic money" OR "cyber currency" OR "peer to peer electronic cash system" OR "bitcoin" OR "ethereum" OR "bitcoin cash" OR "litecoin" OR "ripple" |
| Financial markets intermediary | "stock trading platforms" OR "stock trading app" OR "mobile trading app" OR "investment platform" OR "InvesTech" |
| Information aggregator | "data aggregation platform" OR "data aggregation API" OR "financial comparison portal" OR "financial comparison site" OR "account aggregator app" OR "financial eMarketplace" OR "e-market" |
| Information extractor | "application programming interface" OR "API platform" OR "financial data API" OR "financial API" OR "market data API" |
| Insourcer of sub-processes | "cloud computing" OR "cloud data services" OR "developer API" OR "software as a service" |
| Lending community | "consumer lending" OR "crowd lending" OR "peer to peer lending" OR "P2P lending" OR "social lending" OR "micro lending" OR "person to person lending" OR "peer to peer business lending" OR "P2P business lending" |
| Payment service | *Mobile payments:* "mobile payment service" OR "mobile payment" OR "m-payment" OR "m-pay" OR "mobile money" OR "m-money"  *Online payments:* "digital payments" OR "person­to­person payment" OR "electronic payments" OR "private­to­private payment" OR "P2P payment" OR "digital payment platform" OR "contactless payment system" OR "online payment"  *Billing:* "electronic factoring" OR "digital factoring" OR "online factoring" OR "electronic invoicing" OR "e-invoicing" OR "invoice trading" OR "electronic leasing" OR "digital leasing" OR "online leasing" |
| Robo-advisor | "robo-advisory-services" OR "robo advisor" OR "robo-advisor" OR "robo advice" OR "digital financial advice" |

Table C describes in detail (at the dimension and characteristic level) the empirical iterations (2nd - 7th) carried out during the development process of our final taxonomic structure of FinTech SFs.

Table C. Taxonomy Development Process (Dimension and Characteristic Level)

| Iteration | Development process |
| --- | --- |
| 1^st^ iteration | Definition of the conceptual taxonomic structure. |
| 2^nd^ iteration | In this iteration, according to our conceptual taxonomic structure, we categorized the subset of empirical research articles belonging to the FinTech business model archetype “alternative trading venues” (n=87). Through this first classification, the dimensions (i.e., *D_1_; D_4_; D_12_*) and characteristics that do not describe or are not relevant to at least one of the classified objects (*C_i,j_=* ∅) have been identified and removed (i.e., *C_2,3;_ C_2,4_; C_2,5_; C_2,6_; C_3,1_; C_3,2_; C_3,3_; C_3,6_; C_5,1_; C_5,2_; C_5,4_; C_5,5_; C_5,6_; C_5,7_; C_5,8_; C_8,3_; C_8,4_; C_11,1_; C_11,2_; C_11,3_; C_11,4_; C_11,5_; C_11,6_; C_11,8_; C_11,10_; C_11,12_; C_12,1_; C_12,2_; C_12,3_ C_14,3_)*. Furthermore, the characteristics which describe only one object and are similar in nature have been grouped into higher-level characteristics as follows: 1) marketing plan= *C_5,3_* ∪ *C_5,9_* ∪ *C_6,3_* ∪ *C_6,4_* since our empirical evidence suggests that digital marketing strategies in the FinTech context are fundamentally aimed at aligning sales and marketing resources through alternative revenue models and digital channels to increase their presence and brand´s differentiation/focus vision; and 2) security, privacy, and transparency= *C_8,1_* ∪ *C_10,5_* ∪ *C_10,6_* ∪ *C_11,9_* in view that the existing general provisions regarding these factors contribute to establishing a sustainable FinTech data governance. |
| 3^rd^ Iteration | Subsequently, an additional set of empirical research articles about the FinTech archetype “payment services” (*n=76*) has been incorporated into the analysis. In this iteration, six additional characteristics were relevant for Fintech SFs. These characteristics are displayed within the dimension of “*D_3_* delivery channel” (i.e., *C_3,1_* API; *C_3,2_* mobile applications); “*D_9_* user factors” (i.e., flexibility); “*D_10_* technological factors” (i.e., technology cost); and “*D_11_* value proposition” (i.e., *C_11,3_* convenience/usability; *C_11,4_* customization). Likewise, during this iteration, the characteristics that have a related nature provide a low descriptive power. They were clustered into the higher-level characteristics within “*D_7_* operational factors”: 1) operational alignment= *C_7,2_* ∪ *C_7,3_* given that the degree of alignment between processes and operational policies is a contributing factor in creating an environment that enables FinTechs to achieve optimal operational reliability for reaching their goals; and 2) competency-based human resources= *C_6,6_* ∪ *C_7,7_* ∪ *C_8,2_* ∪ *C_13,1_* considering that the innovation culture impacts the ability of FinTechs to internally develop and retain specialized human resources with comprehensive technical and industry know-how (i.e., domain experts), or the capacity to outsource and manage specialized talent/knowledge. |
| 4^th^ Iteration | During this iteration, we integrated a new set of research articles attributable to the FinTech archetype “insourcer of sub-processes” (*n=20*). Consequently, we identified two new relevant characteristics (i.e., *C_12,2_* infrastructure cost; *C_12,3_* operational cost). Further, since the success factors related to the characteristics within the dimension “*D_3_* delivery channel” mostly refer to the ability to take advantage of diffused knowledge on disruptive innovations to incorporate technical resources such as peer-to-peer networks and mobile applications, we combined the remaining *D_3_* characteristics (i.e., *C_3,1_*; *C_3,2_*; *C_3,4_*; *C_3,5_*; *C_3,7_*) into a characteristic named “technology integration” and relocated it into “*D_10_* technological factors”, thereby merging both dimensions. Additionally, to advance into a more concise taxonomic structure, we combined the characteristics that are interconnected (i.e., cost-benefit dynamic of the innovation= technology profitability (*C_10,4_*) ∪ technology cost; and efficiency= *C_9,9_* ∪ *C_10,8_* ∪ *C_13,2_* within the “operational factors” dimension in order to denote value considerations such as the balance of the practical benefit or worth in the market of a new FinTech product or service in relation to its efficiency or (technology) costs to generate it; operational design= *C_6,7_* ∪ *C_7,1_* ∪ *C_7,4_* ∪ *C_7,5_* and competitive plan= *C_6,2_* ∪ *C_6,5_* ∪ *C_10,1_* ∪ *C_10,2_* ∪ *C_10,7_* within the “strategic factors” dimension for the purpose of consolidating strategic considerations related to the tactic allocation of resources to support regular business routines or the development of a scalable technological infrastructure and capabilities; user socio-economic characteristics= *C_9,1_* ∪ *C_9,2_* within the “user factors” dimension to integrate the characteristics of consumer populations (e.g., intrinsic motivations, shared values) that are important to the development and commercialization of Fintech products and services; and cost structure = *C_12,2_* ∪ *C_12,3_* within a new “economic factors” dimension to assimilate the success factors related to the types and relative proportions of fixed and variable costs of a FinTech company. Afterward, to ensure that each dimension is descriptive by at least containing two characteristics, we incorporated the remaining “*D_8_* and *D_13_* characteristics” (i.e., *C_8,5_* and *C_13,3_*) into the “economic factors” and “operational factors” dimensions, respectively. |
| 5^th^ Iteration | In this iteration, we analyzed the research articles corresponding to the FinTech archetype “lending community” (*n=19*). Based on the analysis of the new set of objects, we merged comparable characteristics (i.e., technology adoption= *C_10,3_* ∪ *C_10,9_* ∪ *C_2,1_* ∪ *C_2,2_* to represent the competence of FinTechs to integrate new technological trends and upgrade existing technologies to make innovative products and services that are well received in the market; market conditions= *C_14,1_* ∪ *C_14,2_* to embody the global market conditions manifested in the capital market, that have an impact on FinTech success; and regulation = *C_14,4_* ∪ *C_14,5_* to concentrate the effects of the legislation imposed by a government to support or regulate economic behaviors in connection with FinTechs. |
| 6^th^ Iteration | Next, we analyzed the articles found for the FinTech archetype “cryptocurrency” (*n=15*). In this iteration, we identified three new characteristics across the dimensions of “*D_10_* technological factors” (i.e., environmental sustainability and ethical issues) and “*D_11_* value proposition” (i.e., disintermediation, *C_11,11_*monetary). Further, we grouped the characteristics of *C_9,7_* usability and flexibility into “ease of use.” Likewise, we conjectured that 1) *C_9,3_* user centricity can be shaped through *C_9,4_* user knowledge, and *C_9,6_* user trust can be formed through *C_9,5_* user satisfaction and *C_7,6_* quality assurances. |
| 7^th^ Iteration | In the last iteration, we examined the objects of the remaining FinTech business model archetypes of “co-creator of financial analysis” (*n=7*), “robo-advisor” (*n=4*), and “information aggregator” (*n=3*). As a result, we identified a new relevant characteristic within the dimension of “*D_11_* value proposition” (i.e., *C_11,12_* decision support). However, no changes were made at the dimensional level. After this iteration, all relevant research articles were classified. Since only a minor change was made to the taxonomic structure, the objective and subjective ending conditions were regarded as fulfilled, thereby ending the development process. At the end of this iteration, we derived a taxonomic structure consisting of seven overarching dimensions: “strategic factors; “operational factors”; “technological factors”; “value proposition”; “user factors”; “economic factors”; and “environmental factors.” Lastly, using this taxonomic structure, we identified the most predominant characteristic within each dimension at the archetype level. |

Table D shows the subjective and objective ending conditions met by each of our performed iterations within the taxonomy development process.

Table D. Ending Conditions met by each Iteration

|  | Iteration - Taxonomic Approach | *1 - conceptual* | *2 - empirical* | *3 - empirical* | *4 - empirical* | *5 - empirical* | *6 - empirical* | *7 - empirical* |
| --- | --- | --- | --- | --- | --- | --- | --- | --- |
|  | **Data Source / Literature** | ***Theoretical Foundation*** | ***Alternative trading venues*** | ***Payment services**** | ***Insourcer of sub-processes*** | ***Lending community*** | ***Crypto-currency*** | ***Others***** |
| Ending Conditions according to Nickerson et al. (2013) | |  |  |  |  |  |  |  |
| *Objective* | |  |  |  |  |  |  |  |
| All objects (or a representative sample) were analyzed | |  | ●  (87) | ●  (163) | ●  (183) | ●  (202) | ●  (217) | ●  (231) |
| No object was merged or split | | ● | ● | ● | ● | ● | ● | ● |
| At least one object is assigned to each characteristic | |  | ● | ● | ● | ● | ● | ● |
| No new dimensions or characteristics were added | |  |  |  |  |  |  | ●^🟄^ |
| No dimensions or characteristics were merged or split | |  |  |  |  |  |  | ● |
| Every dimension is unique | | ● | ● | ● | ● | ● | ● | ● |
| Every characteristic within the dimension is unique | |  |  |  |  | ● | ● | ● |
| *Subjective* | |  |  |  |  |  |  |  |
| *Mutually exclusive:* No object has two different characteristics in a dimension | |  |  |  |  |  | ● | ● |
| *Collectively exhaustive:* Each archetype has at least one characteristic in each dimension | |  |  |  |  | ● | ● | ● |
| *Concise:* Dimensions and characteristics are limited | |  |  |  | ● | ● | ● | ● |
| *Robust:* Sufficient number of dimensions and characteristics | | ● | ● | ● | ● | ● | ● | ● |
| *Comprehensive:* Identification of all (relevant) dimensions of an object | |  |  |  |  |  | ● | ● |
| *Extendable:* Possibility to easily add dimensions and characteristics in the future | | ● | ● | ● | ● | ● | ● | ● |
| *Explanatory:* Dimensions and characteristics sufficiently explain the object | |  |  |  |  | ● | ● | ● |
| *Mutually exclusive:* No object has two different characteristics in a dimension | |  |  |  |  |  | ● | ● |

* Payment services: Online payments, mobile payments, and billing.

** Others: Co-creator of financial analysis, robo-advisory, and information aggregator.

🟄 All relevant research articles were classified, and only a minor change was made to the taxonomic structure.

References for Appendix

Ahlers, G. K., Cumming, D., Günther, C., & Schweizer, D. (2015). Signaling in Equity Crowdfunding. *Entrepreneurship Theory and Practice*, *39*(4), 955–980. https://doi.org/10.1111/etap.12157

Albashrawi, M., & Motiwalla, L. (2019). Privacy and Personalization in Continued Usage Intention of Mobile Banking: An Integrative Perspective. *Information Systems Frontiers*, *21*(5), 1031–1043. https://doi.org/10.1007/s10796-017-9814-7

Albrecht, S., Lutz, B., & Neumann, D. (2020). The behavior of blockchain ventures on Twitter as a determinant for funding success. *Electronic Markets*, *30*(2), 241–257. https://doi.org/10.1007/s12525-019-00371-w

Allison, T. H., Davis, B. C., Short, J. C., & Webb, J. W. (2015). Crowdfunding in a Prosocial Microlending Environment: Examining the Role of Intrinsic versus Extrinsic Cues. *Entrepreneurship Theory and Practice*, *39*(1), 53–73. https://doi.org/10.1111/etap.12108

Allison, T. H., Davis, B. C., Webb, J. W., & Short, J. C. (2017). Persuasion in crowdfunding: An elaboration likelihood model of crowdfunding performance. *Journal of Business Venturing*, *32*(6), 707–725. https://doi.org/10.1016/j.jbusvent.2017.09.002

Alzadjali, K., & Elbanna, A. (2020). Smart Institutional Intervention in the Adoption of Digital Infrastructure: The Case of Government Cloud Computing in Oman. *Information Systems Frontiers*, *22*(2), 365–380. https://doi.org/10.1007/s10796-019-09918-w

Apanasevic, T. (2013). Factors influencing the slow rate of penetration of NFC mobile payment in Western Europe. In *Proceedings of the 2013 International Conference on Mobile Business,* Berlin, Germany.

Arnold, L. G., Booker, B., Dorfleitner, G., & Röhe, M. (2021). Refinancing MFIs with market power: theory and evidence. *Small Business Economics*, *56*(4), 1485–1505. https://doi.org/10.1007/s11187-019-00252-8

Assadi, D., & Hudson, M. (2010). Marketing-Mix of Online Social Lending Websites. *Journal of Electronic Commerce in Organizations*, *8*(3), 15–25. https://doi.org/10.4018/jeco.2010070102

Aswani, R., Kar, A. K., Ilavarasan, P. V., & Dwivedi, Y. K. (2018). Search engine marketing is not all gold: Insights from Twitter and SEOClerks. *International Journal of Information Management*, *38*(1), 107–116. https://doi.org/10.1016/j.ijinfomgt.2017.07.005

Au, Y. A., & Kauffman, R. J. (2008). The economics of mobile payments: Understanding stakeholder issues for an emerging financial technology application. *Electronic Commerce Research and Applications*, *7*(2), 141–164. https://doi.org/10.1016/j.elerap.2006.12.004

Bade, M., & Krezdorn, D. (2018). Cohesion among crowd investors in the presence of moral hazard. *Venture Capital*, *20*(4), 339–353. https://doi.org/10.1080/13691066.2018.1526863

Balocco, R., Ghezzi, A., Bonometti, G., & Renga, F. (2008). Mobile Payment Applications: An Exploratory Analysis of the Italian Diffusion Process. In *2008 7th International Conference on Mobile Business (ICMB),* Barcelona, Spain.

Barkhordari, M., Nourollah, Z., Mashayekhi, H., Mashayekhi, Y., & Ahangar, M. S. (2017). Factors influencing adoption of e-payment systems: an empirical study on Iranian customers. *Information Systems and E-Business Management*, *15*(1), 89–116. https://doi.org/10.1007/s10257-016-0311-1

Beketov, M., Lehmann, K., & Wittke, M. (2018). Robo Advisors: quantitative methods inside the robots. *Journal of Asset Management*, *19*(6), 363–370. https://doi.org/10.1057/s41260-018-0092-9

Belleflamme, P., Lambert, T., & Schwienbacher, A. (2014). Crowdfunding: Tapping the right crowd. *Journal of Business Venturing*, *29*(5), 585–609. https://doi.org/10.1016/j.jbusvent.2013.07.003

Blaschke, M., Riss, U., Haki, K., & Aier, S. (2019). Design principles for digital value co-creation networks: a service-dominant logic perspective. *Electronic Markets*, *29*(3), 443–472. https://doi.org/10.1007/s12525-019-00356-9

Block, J [Jörn], Hornuf, L., & Moritz, A. (2018). Which updates during an equity crowdfunding campaign increase crowd participation? *Small Business Economics*, *50*(1), 3–27. https://doi.org/10.1007/s11187-017-9876-4

Bogusz, C. I., Teigland, R., & Vaast, E. (2019). Designed entrepreneurial legitimacy: the case of a Swedish crowdfunding platform. *European Journal of Information Systems*, *28*(3), 318–335. https://doi.org/10.1080/0960085X.2018.1534039

Bollaert, H., Leboeuf, G., & Schwienbacher, A. (2020). The narcissism of crowdfunding entrepreneurs. *Small Business Economics*, *55*(1), 57–76. https://doi.org/10.1007/s11187-019-00145-w

Bons, R. W. H., Alt, R., Lee, H. G., & Weber, B. (2012). Banking in the Internet and mobile era. *Electronic Markets*, *22*(4), 197–202. https://doi.org/10.1007/s12525-012-0110-6

Bouaynaya, W. (2020). Characterization of Cloud Computing Reversibility as Explored by the DELPHI Method. *Information Systems Frontiers*, *22*(6), 1505–1518. https://doi.org/10.1007/s10796-019-09947-5

Brandt, T., Tian, Y., Hedwig, M., & Neumann, D. (2012). Autonomic management of software as a service systems with multiple quality of service classes. In *Proceedings of the 20th European Conference on Information Systems,* Barcelona, Spain.

Bruckes, M., Westmattelmann, D., Oldeweme, A., & Schewe, G. (2019). Determinants and Barriers of Adopting Robo-Advisory Services. In *Proceedings of the 40th International Conference on Information Systems,* Munich, Germany.

Bruton, G., Khavul, S., Siegel, D., & Wright, M. (2015). New Financial Alternatives in Seeding Entrepreneurship: Microfinance, Crowdfunding, and Peer–to–Peer Innovations. *Entrepreneurship Theory and Practice*, *39*(1), 9–26. https://doi.org/10.1111/etap.12143

Bucherer, E., Eisert, U., & Gassmann, O. (2012). Towards Systematic Business Model Innovation: Lessons from Product Innovation Management. *Creativity and Innovation Management*, *21*(2), 183–198. https://doi.org/10.1111/j.1467-8691.2012.00637.x

Burtch, G., Ghose, A., & Wattal, S. (2013). An Empirical Examination of the Antecedents and Consequences of Contribution Patterns in Crowd-Funded Markets. *Information Systems Research*, *24*(3), 499–519. https://doi.org/10.1287/isre.1120.0468

Burtch, G., Ghose, A., & Wattal, S. (2014). Cultural Differences and Geography as Determinants of Online Prosocial Lending. *MIS Quarterly*, *38*(3), 773–794. https://www.jstor.org/stable/26634995

Burtch, G., Ghose, A., & Wattal, S. (2016). Secret Admirers: An Empirical Examination of Information Hiding and Contribution Dynamics in Online Crowdfunding. *Information Systems Research*, *27*(3), 478–496. https://doi.org/10.1287/isre.2016.0642

Butticè, V., Colombo, M. G., & Wright, M. (2017). Serial Crowdfunding, Social Capital, and Project Success. *Entrepreneurship Theory and Practice*, *41*(2), 183–207. https://doi.org/10.1111/etap.12271

Buyya, R., Srirama, S. N., Casale, G., Calheiros, R., Simmhan, Y., Varghese, B., Gelenbe, E., Javadi, B., Vaquero, L. M., Netto, M. A. S., Toosi, A. N., Rodriguez, M. A., Llorente, I. M., Di Vimercati, S. D. C., Samarati, P., Milojicic, D., Varela, C., Bahsoon, R., Assuncao, M. D. de, . . . Shen, H. (2019). A Manifesto for Future Generation Cloud Computing. *ACM Computing Surveys*, *51*(5), 1–38. https://doi.org/10.1145/3241737

Cai, J., Walkling, R. A., & Yang, K. (2016). The Price of Street Friends: Social Networks, Informed Trading, and Shareholder Costs. *Journal of Financial and Quantitative Analysis*, *51*(3), 801–837. https://doi.org/10.1017/S0022109016000399

Chan, C. R., Park, H. D., Patel, P., & Gomulya, D. (2018). Reward-based crowdfunding success: decomposition of the project, product category, entrepreneur, and location effects. *Venture Capital*, *20*(3), 285–307. https://doi.org/10.1080/13691066.2018.1480267

Chan, C. S. R., & Parhankangas, A. (2017). Crowdfunding Innovative Ideas: How Incremental and Radical Innovativeness Influence Funding Outcomes. *Entrepreneurship Theory and Practice*, *41*(2), 237–263. https://doi.org/10.1111/etap.12268

Chen, D [Dongyu], Lai, F., & Lin, Z [Zhangxi] (2014). A trust model for online peer-to-peer lending: a lender’s perspective. *Information Technology and Management*, *15*(4), 239–254. https://doi.org/10.1007/s10799-014-0187-z

Chen, D [Dongyu], Li, X [Xiaolin], & Lai, F. (2017). Gender discrimination in online peer-to-peer credit lending: evidence from a lending platform in China. *Electronic Commerce Research*, *17*(4), 553–583. https://doi.org/10.1007/s10660-016-9247-2

Chen, Q., Li, J.‑W., Liu, J.‑G., Han, J.‑T., Shi, Y., & Guo, X.‑H. (2021). Borrower Learning Effects: Do Prior Experiences Promote Continuous Successes in Peer-to-Peer Lending? *Information Systems Frontiers*, *23*(4), 963–986. https://doi.org/10.1007/s10796-020-10006-7

Chen, X., Jin, F., Zhang, Q., & Yang, L. (2016). Are investors rational or perceptual in P2P lending? *Information Systems and E-Business Management*, *14*(4), 921–944. https://doi.org/10.1007/s10257-016-0305-z

Cholakova, M., & Clarysse, B. (2015). Does the Possibility to Make Equity Investments in Crowdfunding Projects Crowd Out Reward–Based Investments? *Entrepreneurship Theory and Practice*, *39*(1), 145–172. https://doi.org/10.1111/etap.12139

Chou, S.‑W., & Chiang, C.‑H. (2013). Understanding the formation of software-as-a-service (SaaS) satisfaction from the perspective of service quality. *Decision Support Systems*, *56*, 148–155. https://doi.org/10.1016/j.dss.2013.05.013

Chun, S.‑H., Cho, W., & Subramanyam, R. (2016). Transaction security investments in online marketplaces: An analytical examination of financial liabilities. *Decision Support Systems*, *92*, 91–102. https://doi.org/10.1016/j.dss.2016.09.015

CLAUSS, T., NIEMAND, T., KRAUS, S., SCHNETZER, P., & BREM, A. (2020). INCREASING CROWDFUNDING SUCCESS THROUGH SOCIAL MEDIA: THE IMPORTANCE OF REACH AND UTILISATION IN REWARD-BASED CROWDFUNDING. *International Journal of Innovation Management*, *24*(03), 2050026. https://doi.org/10.1142/S1363919620500267

Colombo, M. G., Franzoni, C., & Rossi–Lamastra, C. (2015). Internal Social Capital and the Attraction of Early Contributions in Crowdfunding. *Entrepreneurship Theory and Practice*, *39*(1), 75–100. https://doi.org/10.1111/etap.12118

Courtney, C., Dutta, S., & Li, Y. (2017). Resolving Information Asymmetry: Signaling, Endorsement, and Crowdfunding Success. *Entrepreneurship Theory and Practice*, *41*(2), 265–290. https://doi.org/10.1111/etap.12267

Cousins, K., Subramanian, H., & Esmaeilzadeh, P. (2019). A Value-sensitive Design Perspective of Cryptocurrencies: A Research Agenda. *Communications of the Association for Information Systems*, 511–547. https://doi.org/10.17705/1CAIS.04527

Crosetto, P., & Regner, T. (2018). It's never too late: Funding dynamics and self pledges in reward-based crowdfunding. *Research Policy*, *47*(8), 1463–1477. https://doi.org/10.1016/j.respol.2018.04.020

Culkin, N., Murzacheva, E., & Davis, A. (2016). Critical innovations in the UK peer-to-peer (P2P) and equity alternative finance markets for small firm growth. *The International Journal of Entrepreneurship and Innovation*, *17*(3), 194–202. https://doi.org/10.1177/1465750316655906

Cumming, D., Meoli, M., & Vismara, S. (2021). Does equity crowdfunding democratize entrepreneurial finance? *Small Business Economics*, *56*(2), 533–552. https://doi.org/10.1007/s11187-019-00188-z

Cummings, M. E., Rawhouser, H., Vismara, S., & Hamilton, E. L. (2020). An equity crowdfunding research agenda: evidence from stakeholder participation in the rulemaking process. *Small Business Economics*, *54*(4), 907–932. https://doi.org/10.1007/s11187-018-00134-5

Cusumano, M. A. (2014). The Bitcoin ecosystem. *Communications of the ACM*, *57*(10), 22–24. https://doi.org/10.1145/2661047

Cuylen, A., Kosch, L., & Breitner, M. H. (2016). Development of a maturity model for electronic invoice processes. *Electronic Markets*, *26*(2), 115–127. https://doi.org/10.1007/s12525-015-0206-x

Dahlberg, T., Bouwman, H., Cerpa, N., & Guo, J [Jie] (2015). M-Payment - How Disruptive Technologies Could Change The Payment Ecosystem. In *Proceedings of the 23rd European Conference on Information Systems,* Münster, Germany.

Dai, H., & Salam, A. F. (2014). Does service convenience matter? An empirical assessment of service quality, service convenience and exchange relationship in electronic mediated environment. *Electronic Markets*, *24*(4), 269–284. https://doi.org/10.1007/s12525-014-0170-x

David-West, O., Iheanachor, N., & Kelikume, I. (2018). A resource-based view of digital financial services (DFS): An exploratory study of Nigerian providers. *Journal of Business Research*, *88*, 513–526. https://doi.org/10.1016/j.jbusres.2018.01.034

Davis, B. C., Hmieleski, K. M., Webb, J. W., & Coombs, J. E. (2017). Funders' positive affective reactions to entrepreneurs' crowdfunding pitches: The influence of perceived product creativity and entrepreneurial passion. *Journal of Business Venturing*, *32*(1), 90–106. https://doi.org/10.1016/j.jbusvent.2016.10.006

de Reuver, M., Blok, S., & Bowman, H. (2015). Stakeholder Preferences for Mobile Payment Security Platforms: Understanding Trade-offs Between SIM, Embedded and Cloud-based Secure Elements. In *Proceedings of the 2015 International Conference on Mobile Business,* Forth Worth, TX, USA.

Derks, J., Gordijn, J., & Siegmann, A. (2018). From chaining blocks to breaking even: A study on the profitability of bitcoin mining from 2012 to 2016. *Electronic Markets*, *28*(3), 321–338. https://doi.org/10.1007/s12525-018-0308-3

Dizdarević, J., Carpio, F., Jukan, A., & Masip-Bruin, X. (2019). A Survey of Communication Protocols for Internet of Things and Related Challenges of Fog and Cloud Computing Integration. *ACM Computing Surveys*, *51*(6), 1–29. https://doi.org/10.1145/3292674

Du, K. (2018). Complacency, capabilities, and institutional pressure: understanding financial institutions’ participation in the nascent mobile payments ecosystem. *Electronic Markets*, *28*(3), 307–319. https://doi.org/10.1007/s12525-017-0267-0

Du, Z., Li, M., & Wang, K. (2019). “The more options, the better?” Investigating the impact of the number of options on backers’ decisions in reward-based crowdfunding projects. *Information & Management*, *56*(3), 429–444. https://doi.org/10.1016/j.im.2018.08.003

Du, Z., Wang, K., & Li, M. (2019). Promoting crowdfunding with lottery: The impact on campaign performance. *Information & Management*, *56*(8), 103159. https://doi.org/10.1016/j.im.2019.04.002

Efrat, K., & Gilboa, S. (2020). Relationship approach to crowdfunding: how creators and supporters interaction enhances projects’ success. *Electronic Markets*, *30*(4), 899–911. https://doi.org/10.1007/s12525-019-00391-6

Eldridge, D., Nisar, T. M., & Torchia, M. (2021). What impact does equity crowdfunding have on SME innovation and growth? An empirical study. *Small Business Economics*, *56*(1), 105–120. https://doi.org/10.1007/s11187-019-00210-4

El-Haddadeh, R. (2020). Digital Innovation Dynamics Influence on Organisational Adoption: The Case of Cloud Computing Services. *Information Systems Frontiers*, *22*(4), 985–999. https://doi.org/10.1007/s10796-019-09912-2

Eyal, I., & Sirer, E. G. (2018). Majority is not enough. *Communications of the ACM*, *61*(7), 95–102. https://doi.org/10.1145/3212998

Feller, J., Gleasure, R., & Treacy, S. (2017). Information Sharing and User Behavior in Internet-enabled Peer-to-peer Lending Systems: An Empirical Study. *Journal of Information Technology*, *32*(2), 127–146. https://doi.org/10.1057/jit.2016.1

Feola, R., Vesci, M., Marinato, E., & Parente, R. (2021). Segmenting “digital investors”: evidence from the Italian equity crowdfunding market. *Small Business Economics*, *56*(3), 1235–1250. https://doi.org/10.1007/s11187-019-00265-3

Ferri, L., Spanò, R., & Tomo, A. (2020). Cloud computing in high tech startups: evidence from a case study. *Technology Analysis & Strategic Management*, *32*(2), 146–157. https://doi.org/10.1080/09537325.2019.1641594

Fisch, C., Masiak, C., Vismara, S., & Block, J. (2021). Motives and profiles of ICO investors. *Journal of Business Research*, *125*, 564–576. https://doi.org/10.1016/j.jbusres.2019.07.036

Foster, C., & Heeks, R. (2013). Innovation and Scaling of ICT for the Bottom-Of-The-Pyramid. *Journal of Information Technology*, *28*(4), 296–315. https://doi.org/10.1057/jit.2013.19

Gao, Y., Yu, S.‑H., & Shiue, Y.‑C. (2018). The performance of the P2P finance industry in China. *Electronic Commerce Research and Applications*, *30*, 138–148. https://doi.org/10.1016/j.elerap.2018.06.002

Garrison, G., Kim, S., & Wakefield, R. L. (2012). Success factors for deploying cloud computing. *Communications of the ACM*, *55*(9), 62–68. https://doi.org/10.1145/2330667.2330685

Garrison, G., Wakefield, R. L., & Kim, S. (2015). The effects of IT capabilities and delivery model on cloud computing success and firm performance for cloud supported processes and operations. *International Journal of Information Management*, *35*(4), 377–393. https://doi.org/10.1016/j.ijinfomgt.2015.03.001

Genkin, D., Papadopoulos, D., & Papamanthou, C. (2018). Privacy in decentralized cryptocurrencies. *Communications of the ACM*, *61*(6), 78–88. https://doi.org/10.1145/3132696

Gerber, E. M., & Hui, J. (2013). Crowdfunding - Motivations and deterrents for participation. *ACM Transactions on Computer-Human Interaction*, *20*(6), 1–32. https://doi.org/10.1145/2530540

Gill, S. S., & Buyya, R. (2019). A Taxonomy and Future Directions for Sustainable Cloud Computing. *ACM Computing Surveys*, *51*(5), 1–33. https://doi.org/10.1145/3241038

Giudici, G., Guerini, M., & Rossi-Lamastra, C. (2018). Reward-based crowdfunding of entrepreneurial projects: the effect of local altruism and localized social capital on proponents’ success. *Small Business Economics*, *50*(2), 307–324. https://doi.org/10.1007/s11187-016-9830-x

Gleasure, R., & Feller, J. (2016). Does Heart or Head Rule Donor Behaviors in Charitable Crowdfunding Markets? *International Journal of Electronic Commerce*, *20*(4), 499–524. https://doi.org/10.1080/10864415.2016.1171975

Gleasure, R., O'Reilly, P., & Cahalane, M. (2017). Inclusive Technologies, Selective Traditions: A Socio-material Case Study of Crowdfunded Book Publishing. *Journal of Information Technology*, *32*(4), 326–343. https://doi.org/10.1057/s41265-017-0041-y

Gobble, M. M. (2012). Everyone Is a Venture Capitalist: The New Age of Crowdfunding. *Research Technology Management*, *55*(4), 4–7. https://www.jstor.org/stable/26586617

Gomber, P., Kauffman, R. J., Parker, C., & Weber, B. W. (2018). On the Fintech Revolution: Interpreting the Forces of Innovation, Disruption, and Transformation in Financial Services. *Journal of Management Information Systems*, *35*(1), 220–265. https://doi.org/10.1080/07421222.2018.1440766

Gozman, D., Liebenau, J., & Mangan, J. (2018). The Innovation Mechanisms of Fintech Start-Ups: Insights from SWIFT’s Innotribe Competition. *Journal of Management Information Systems*, *35*(1), 145–179. https://doi.org/10.1080/07421222.2018.1440768

Guo, J [Jingzhi], Chow, A., & Wigand, R. T. (2011). Virtual wealth protection through virtual money exchange. *Electronic Commerce Research and Applications*, *10*(3), 313–330. https://doi.org/10.1016/j.elerap.2010.10.003

Haas, P., & Blohm, I. (2017). Blueprinting Crowdfunding - Designing a Crowdfunding Service Configuration Framework. In *Proceedings of the 13th International Conference on Wirtschaftsinformatik,* St. Gallen, Switzerland.

Haddad, C., & Hornuf, L. (2019). The emergence of the global fintech market: economic and technological determinants. *Small Business Economics*, *53*(1), 81–105. https://doi.org/10.1007/s11187-018-9991-x

Hashim, M. J., Kannan, K. N., & Maximiano, S. (2017). Information Feedback, Targeting, and Coordination: An Experimental Study. *Information Systems Research*, *28*(2), 289–308. https://doi.org/10.1287/isre.2016.0663

Hedman, J., & Henningsson, S. (2012). Competition and collaboration shaping the digital payment infrastructure. In *ICEC '12: Fourteenth International Conference on Electronic Commerce,* Singapore Singapore.

Henning-Thurau, T., Sattler, H., Dyckhoff, H., Franke, N., & Schreyögg, G. (2023). *VHB-JOURQUAL3*. https://www.vhbonline.org/vhb4you/vhb-jourqual/vhb-jourqual-3/gesamtliste

Hillman, S., Neustaedter, C., Oduor, E., & Pang, C. (2014). User challenges and successes with mobile payment services in North America. In *Proceedings of the 16th International Conference on Human-Computer Interaction with Mobile Devices & Services,* Toronto, Canada.

Hjelholt, M., & Damsgaard, J. (2012). The genesis and evolution of digital payment platforms. In *Proceedings of the 20th European Conference on Information Systems,* Barcelona, Spain.

Hoegen, A., Steininger, D. M., & Veit, D. (2018). How do investors decide? An interdisciplinary review of decision-making in crowdfunding. *Electronic Markets*, *28*(3), 339–365. https://doi.org/10.1007/s12525-017-0269-y

Hong, I. B., & Cha, H. S. (2013). The mediating role of consumer trust in an online merchant in predicting purchase intention. *International Journal of Information Management*, *33*(6), 927–939. https://doi.org/10.1016/j.ijinfomgt.2013.08.007

Hornuf, L., & Neuenkirch, M. (2017). Pricing shares in equity crowdfunding. *Small Business Economics*, *48*(4), 795–811. https://doi.org/10.1007/s11187-016-9807-9

Hornuf, L., & Schwienbacher, A. (2017). Should securities regulation promote equity crowdfunding? *Small Business Economics*, *49*(3), 579–593. https://doi.org/10.1007/s11187-017-9839-9

Huang, C.‑K., Wang, T [Tawei], & Huang, T.‑Y. (2020). Initial Evidence on the Impact of Big Data Implementation on Firm Performance. *Information Systems Frontiers*, *22*(2), 475–487. https://doi.org/10.1007/s10796-018-9872-5

Huang, W., Meoli, M., & Vismara, S. (2020). The geography of initial coin offerings. *Small Business Economics*, *55*(1), 77–102. https://doi.org/10.1007/s11187-019-00135-y

Iman, N. (2018). Is mobile payment still relevant in the fintech era? *Electronic Commerce Research and Applications*, *30*, 72–82. https://doi.org/10.1016/j.elerap.2018.05.009

Islam, A., Muzi, S., & Rodriguez Meza, J. L. (2018). Does mobile money use increase firms’ investment? Evidence from Enterprise Surveys in Kenya, Uganda, and Tanzania. *Small Business Economics*, *51*(3), 687–708. https://doi.org/10.1007/s11187-017-9951-x

Jagtiani, J., & Lemieux, C. (2018). Do fintech lenders penetrate areas that are underserved by traditional banks? *Journal of Economics and Business*, *100*, 43–54. https://doi.org/10.1016/j.jeconbus.2018.03.001

Jaspers, F., Prencipe, A., & Ende, J. (2012). Organizing Interindustry Architectural Innovations: Evidence from Mobile Communication Applications. *Journal of Product Innovation Management*, *29*(3), 419–431. https://doi.org/10.1111/j.1540-5885.2012.00915.x

Johnson, S [Susan], Ashta, A., & Assadi, D. (2010). Online or Offline? The rise of “peer-to-peer” lending in microfinance. *Journal of Electronic Commerce in Organizations*, *8*(3), 26–37. https://doi.org/10.4018/jeco.2010070103

Josefy, M., Dean, T. J., Albert, L. S., & Fitza, M. A. (2017). The Role of Community in Crowdfunding Success: Evidence on Cultural Attributes in Funding Campaigns to “Save the Local Theater”. *Entrepreneurship Theory and Practice*, *41*(2), 161–182. https://doi.org/10.1111/etap.12263

Jung, D., Dorner, V., Weinhardt, C., & Pusmaz, H. (2018). Designing a robo-advisor for risk-averse, low-budget consumers. *Electronic Markets*, *28*(3), 367–380. https://doi.org/10.1007/s12525-017-0279-9

Kang, L., Jiang, Q., & Tan, C.‑H. (2017). Remarkable advocates: An investigation of geographic distance and social capital for crowdfunding. *Information & Management*, *54*(3), 336–348. https://doi.org/10.1016/j.im.2016.09.001

Karjaluoto, H., Shaikh, A. A., Saarijärvi, H., & Saraniemi, S. (2019). How perceived value drives the use of mobile financial services apps. *International Journal of Information Management*, *47*, 252–261. https://doi.org/10.1016/j.ijinfomgt.2018.08.014

Kauffman, R. J., Ma, D., & Yu, M. (2018). A metrics suite of cloud computing adoption readiness. *Electronic Markets*, *28*(1), 11–37. https://doi.org/10.1007/s12525-015-0213-y

Kazan, E. (2015). The Innovative Capabilities Of Digital Payment Platforms: A Comparative Study Of Apple Pay & Google Wallet. In *Proceedings of the 2015 International Conference on Mobile Business,* Forth Worth, TX, USA.

Kazan, E., & Damsgaard, J. (2014). An investigation of digital payment platform designs: A comparative study of four European solutions. In *Proceedings of the 22nd European Conference on Information Systems,* Tel Aviv, Israel.

Kazan, E., & Damsgaard, J. (2016). Towards A New Market Entry Framework For Digital Payment Platforms. *Communications of the Association for Information Systems*, *38*, 761–783. https://doi.org/10.17705/1CAIS.03837

Kazan, E., Tan, C.‑W., & Lim, E. (2015). Disentangling Competition Among Platform Driven Strategic Groups: A Comparative Case Study Of Uk Mobile Payment Platforms. In *Proceedings of the 2015 International Conference on Mobile Business,* Forth Worth, TX, USA.

Kgoroeadira, R., Burke, A., & van Stel, A. (2019). Small business online loan crowdfunding: who gets funded and what determines the rate of interest? *Small Business Economics*, *52*(1), 67–87. https://doi.org/10.1007/s11187-017-9986-z

Kher, R., Terjesen, S., & Liu, C. (2021). Blockchain, Bitcoin, and ICOs: a review and research agenda. *Small Business Economics*, *56*(4), 1699–1720. https://doi.org/10.1007/s11187-019-00286-y

Kim, C., Tao, W., Shin, N., & Kim, K.‑S. (2010). An empirical study of customers’ perceptions of security and trust in e-payment systems. *Electronic Commerce Research and Applications*, *9*(1), 84–95. https://doi.org/10.1016/j.elerap.2009.04.014

Kim, D. J., Yim, M.‑S., Sugumaran, V., & Rao, H. R. (2016). Web assurance seal services, trust and consumers’ concerns: an investigation of e-commerce transaction intentions across two nations. *European Journal of Information Systems*, *25*(3), 252–273. https://doi.org/10.1057/ejis.2015.16

Kleinert, S., Volkmann, C., & Grünhagen, M. (2020). Third-party signals in equity crowdfunding: the role of prior financing. *Small Business Economics*, *54*(1), 341–365. https://doi.org/10.1007/s11187-018-0125-2

Koch, J.‑A., & Siering, M. (2015). Crowdfunding success factors: The characteristics of successfully funded projects on crowdfunding platforms. In *Proceedings of the 23rd European Conference on Information Systems,* Münster, Germany.

Koch, J.‑A., & Siering, M. (2019). The recipe of successful crowdfunding campaigns. *Electronic Markets*, *29*(4), 661–679. https://doi.org/10.1007/s12525-019-00357-8

Kromidha, E., & Robson, P. (2016). Social identity and signalling success factors in online crowdfunding. *Entrepreneurship & Regional Development*, *28*(9-10), 605–629. https://doi.org/10.1080/08985626.2016.1198425

Kugler, L. (2018). Why cryptocurrencies use so much energy. *Communications of the ACM*, *61*(7), 15–17. https://doi.org/10.1145/3213762

Kumar, P., & Kumar, R. (2019). Issues and Challenges of Load Balancing Techniques in Cloud Computing. *ACM Computing Surveys*, *51*(6), 1–35. https://doi.org/10.1145/3281010

Kunz, M. M., Bretschneider, U., Erler, M., & Leimeister, J. M. (2017). An empirical investigation of signaling in reward-based crowdfunding. *Electronic Commerce Research*, *17*(3), 425–461. https://doi.org/10.1007/s10660-016-9249-0

Kuppuswamy, V., & Bayus, B. L. (2017). Does my contribution to your crowdfunding project matter? *Journal of Business Venturing*, *32*(1), 72–89. https://doi.org/10.1016/j.jbusvent.2016.10.004

Lang, M., Wiesche, M., & Krcmar, H. (2018). Criteria for Selecting Cloud Service Providers: A Delphi Study of Quality-of-Service Attributes. *Information & Management*, *55*(6), 746–758. https://doi.org/10.1016/j.im.2018.03.004

Lashitew, A. A., van Tulder, R., & Liasse, Y. (2019). Mobile phones for financial inclusion: What explains the diffusion of mobile money innovations? *Research Policy*, *48*(5), 1201–1215. https://doi.org/10.1016/j.respol.2018.12.010

Lee, C. H., & Bian, Y. (2018). Factors Affecting Success of Serial Crowdfunding: From Heuristic and Systematic Perspectives. In *Proceedings of the 22nd Pacific Asia Conference on Information Systems,* Tokio, Japan.

Lee, S., Park, S. B., & Lim, G. G. (2013). Using balanced scorecards for the evaluation of “Software-as-a-service”. *Information & Management*, *50*(7), 553–561. https://doi.org/10.1016/j.im.2013.07.006

Lehner, O. M. (2014). The formation and interplay of social capital in crowdfunded social ventures. *Entrepreneurship & Regional Development*, *26*(5-6), 478–499. https://doi.org/10.1080/08985626.2014.922623

Li, X [Xin], & Wang, C. A. (2017). The technology and economic determinants of cryptocurrency exchange rates: The case of Bitcoin. *Decision Support Systems*, *95*, 49–60. https://doi.org/10.1016/j.dss.2016.12.001

Li, Y.‑M., Liou, J.‑H., & Li, Y.‑W. (2020). A social recommendation approach for reward-based crowdfunding campaigns. *Information & Management*, *57*(7), 103246. https://doi.org/10.1016/j.im.2019.103246

Liang, K., Jiang, C., Lin, Z [Zhangxi], Ning, W., & Jia, Z. (2017). The nature of sellers’ cyber credit in C2C e-commerce: the perspective of social capital. *Electronic Commerce Research*, *17*(1), 133–147. https://doi.org/10.1007/s10660-016-9231-x

Liébana-Cabanillas, F., Muñoz-Leiva, F., & Sánchez-Fernández, J. (2015). Behavioral Model of Younger Users in M-Payment Systems. *Journal of Organizational Computing and Electronic Commerce*, *25*(2), 169–190. https://doi.org/10.1080/10919392.2015.1033947

Lin, J., Wang, B., Wang, N [Na], & Lu, Y. (2014). Understanding the evolution of consumer trust in mobile commerce: a longitudinal study. *Information Technology and Management*, *15*(1), 37–49. https://doi.org/10.1007/s10799-013-0172-y

Liu, D., Brass, D. J., Yong, L., & Chen, D [Donyu] (2015). Friendship in online peer-to-peer lending: Pipes, prisms, and relational herding. *MIS Quarterly*, *39*(3), 729–742.

Liu, J., Kauffman, R. J., & Ma, D. (2015). Competition, cooperation, and regulation: Understanding the evolution of the mobile payments technology ecosystem. *Electronic Commerce Research and Applications*, *14*(5), 372–391. https://doi.org/10.1016/j.elerap.2015.03.003

Liu, Y [Y.], Han, H., & DeBello, J. (2018). The challenges of business analytics: Successes and failures. In *Proceedings of the 51st Hawaii International Conference on System Sciences* (pp. 840–850).

Liu, Y [Yong], Wang, M., Huang, D., Huang, Q., Yang, H., & Li, Z. (2019). The impact of mobility, risk, and cost on the users’ intention to adopt mobile payments. *Information Systems and E-Business Management*, *17*(2-4), 319–342. https://doi.org/10.1007/s10257-019-00449-0

Löher, J., Schneck, S., & Werner, A. (2018). A research note on entrepreneurs’ financial commitment and crowdfunding success. *Venture Capital*, *20*(3), 309–322. https://doi.org/10.1080/13691066.2018.1480864

Loukis, E., Janssen, M., & Mintchev, I. (2019). Determinants of software-as-a-service benefits and impact on firm performance. *Decision Support Systems*, *117*, 38–47. https://doi.org/10.1016/j.dss.2018.12.005

Lu, Y., Yang, S., Chau, P. Y., & Cao, Y. (2011). Dynamics between the trust transfer process and intention to use mobile payment services: A cross-environment perspective. *Information & Management*, *48*(8), 393–403. https://doi.org/10.1016/j.im.2011.09.006

Lukkarinen, A., Teich, J. E., Wallenius, H., & Wallenius, J. (2016). Success drivers of online equity crowdfunding campaigns. *Decision Support Systems*, *87*, 26–38. https://doi.org/10.1016/j.dss.2016.04.006

Ma, L., Zhao, X., Zhou, Z., & Liu, Y [Yuanyuan] (2018). A new aspect on P2P online lending default prediction using meta-level phone usage data in China. *Decision Support Systems*, *111*, 60–71. https://doi.org/10.1016/j.dss.2018.05.001

Mai, F., Shan, Z., Bai, Q., Wang, X., & Chiang, R. H. (2018). How Does Social Media Impact Bitcoin Value? A Test of the Silent Majority Hypothesis. *Journal of Management Information Systems*, *35*(1), 19–52. https://doi.org/10.1080/07421222.2018.1440774

Mamonov, S., & Malaga, R. (2018). Success factors in Title III equity crowdfunding in the United States. *Electronic Commerce Research and Applications*, *27*, 65–73. https://doi.org/10.1016/j.elerap.2017.12.001

Markendahl, J. (2013). Change of Market Structure for Mobile Payments Services in Sweden - The Case of SMS Tickets. In *Proceedings of the 2013 International Conference on Mobile Business,* Berlin, Germany.

Masiak, C., Block, J. H., Masiak, T., Neuenkirch, M., & Pielen, K. N. (2020). Initial coin offerings (ICOs): market cycles and relationship with bitcoin and ether. *Small Business Economics*, *55*(4), 1113–1130. https://doi.org/10.1007/s11187-019-00176-3

Meiklejohn, S., Pomarole, M., Jordan, G., Levchenko, K., McCoy, D., Voelker, G. M., & Savage, S. (2013). A fistful of bitcoins. In *Proceedings of the 2013 Conference on Internet Measurement Conference,* Barcelona, Spain.

Mendoza-Tello, J. C., Mora, H., Pujol-López, F. A., & Lytras, M. D. (2019). Disruptive innovation of cryptocurrencies in consumer acceptance and trust. *Information Systems and E-Business Management*, *17*(2-4), 195–222. https://doi.org/10.1007/s10257-019-00415-w

Miglo, A., & Miglo, V. (2019). Market imperfections and crowdfunding. *Small Business Economics*, *53*(1), 51–79. https://doi.org/10.1007/s11187-018-0037-1

Mikalef, P., Krogstie, J., Van de Wetering, R., & Pappas, I. (2018). A stage model for uncovering inertia in big data analytics adoption. In *Proceedings of the 22nd Pacific Asia Conference on Information Systems,* Tokio, Japan.

Mikalef, P., Pappas, I. O., Krogstie, J., & Giannakos, M. (2018). Big data analytics capabilities: a systematic literature review and research agenda. *Information Systems and E-Business Management*, *16*(3), 547–578. https://doi.org/10.1007/s10257-017-0362-y

Mitra, S., & Euchner, J. (2016). Business Acceleration at Scale: An Interview with Sramana Mitra. *Research Technology Management*, *59*(3), 12–20. https://doi.org/10.1080/08956308.2016.1161398

Mohammadi, A., & Shafi, K. (2018). Gender differences in the contribution patterns of equity-crowdfunding investors. *Small Business Economics*, *50*(2), 275–287. https://doi.org/10.1007/s11187-016-9825-7

Mollick, E. (2014). The dynamics of crowdfunding: An exploratory study. *Journal of Business Venturing*, *29*(1), 1–16. https://doi.org/10.1016/j.jbusvent.2013.06.005

Momtaz, P. P. (2021a). Entrepreneurial Finance and Moral Hazard: Evidence from Token Offerings. *Journal of Business Venturing*, *36*(5), 106001. https://doi.org/10.1016/j.jbusvent.2020.106001

Momtaz, P. P. (2021b). Initial coin offerings, asymmetric information, and loyal CEOs. *Small Business Economics*, *57*(2), 975–997. https://doi.org/10.1007/s11187-020-00335-x

Moroni, A., Talamo, M., & Dimitri, A. (2015). Adoption factors of NFC Mobile Proximity Payments in Italy. In *Proceedings of the 17th International Conference on Human-Computer Interaction with Mobile Devices and Services,* Copenhagen, Denmark.

Mou, J., Shin, D.‑H., & Cohen, J. F. (2017). Trust and risk in consumer acceptance of e-services. *Electronic Commerce Research*, *17*(2), 255–288. https://doi.org/10.1007/s10660-015-9205-4

Narayanan, A., & Clark, J. (2017). Bitcoin's academic pedigree. *Communications of the ACM*, *60*(12), 36–45. https://doi.org/10.1145/3132259

Nickerson, R. C., Varshney, U., & Muntermann, J. (2013). A method for taxonomy development and its application in information systems. *European Journal of Information Systems*, *22*(3), 336–359. https://doi.org/10.1057/ejis.2012.26

O’Reilly, P., Duane, A., & Andreev, P. (2012). To M-Pay or not to M-Pay—Realising the potential of smart phones: conceptual modeling and empirical validation. *Electronic Markets*, *22*(4), 229–241. https://doi.org/10.1007/s12525-012-0105-3

Ogbanufe, O., & Kim, D. J. (2018). Comparing fingerprint-based biometrics authentication versus traditional authentication methods for e-payment. *Decision Support Systems*, *106*, 1–14. https://doi.org/10.1016/j.dss.2017.11.003

Ondrus, J [J.], Lyytinnen, K., & Pigneur, Y [Y.]. (2009). Why mobile payments fail? Towards a dynamic and multi-perspective explanation. In *Proceedings of the 42nd Hawaii International Conference on System Sciences* (pp. 1–10).

Ondrus, J [Jan] (2015). Clashing over the NFC Secure Element for Platform Leadership in the Mobile Payment Ecosystem. In *ICEC '15: The 17th International Conference on Electronic Commerce 2015,* Seoul Republic of Korea.

Ondrus, J [Jan], Gannamaneni, A., & Lyytinen, K. (2015). The Impact of Openness on the Market Potential of Multi-Sided Platforms: A Case Study of Mobile Payment Platforms. *Journal of Information Technology*, *30*(3), 260–275. https://doi.org/10.1057/jit.2015.7

Ondrus, J [Jan], & Lyytinen, K. (2011). Mobile Payments Market: Towards Another Clash of the Titans? In *2011 Tenth International Conference on Mobile Business, ICMB,* Como, Italy.

Ondrus, J [Jan], & Pigneur, Y [Yves] (2009). Near field communication: an assessment for future payment systems. *Information Systems and E-Business Management*, *7*(3), 347–361. https://doi.org/10.1007/s10257-008-0093-1

O'Reilly, P., & Finnegan, P. (2010). Intermediaries in inter-organisational networks: building a theory of electronic marketplace performance. *European Journal of Information Systems*, *19*(4), 462–480. https://doi.org/10.1057/ejis.2010.12

Ozcan, P., & Santos, F. M. (2015). The market that never was: Turf wars and failed alliances in mobile payments. *Strategic Management Journal*, *36*(10), 1486–1512. https://doi.org/10.1002/smj.2292

Parhankangas, A., & Renko, M. (2017). Linguistic style and crowdfunding success among social and commercial entrepreneurs. *Journal of Business Venturing*, *32*(2), 215–236. https://doi.org/10.1016/j.jbusvent.2016.11.001

Park, H. M., & Islam, M. T. (2014). The potential and limitations of a public mobile payment service: Did Bangladesh electronic money transfer system make a difference in “unbanked” communities? In *Proceedings of the 18th Pacific Asia Conference on Information Systems,* Chengdu, China.

Pastoll, C., Rochwerg, T., Vlaar, B., & Compeau, D. (2014). Starbucks Canada: The mobile payments decision. In *Proceedings of the 35th International Conference on Information Systems,* Auckland, New Zealand.

Piva, E., & Rossi-Lamastra, C. (2018). Human capital signals and entrepreneurs’ success in equity crowdfunding. *Small Business Economics*, *51*(3), 667–686. https://doi.org/10.1007/s11187-017-9950-y

Polzin, F., Toxopeus, H., & Stam, E. (2018). The wisdom of the crowd in funding: information heterogeneity and social networks of crowdfunders. *Small Business Economics*, *50*(2), 251–273. https://doi.org/10.1007/s11187-016-9829-3

Pousttchi, K., Schiessler, M., & Wiedemann, D. G. (2009). Proposing a comprehensive framework for analysis and engineering of mobile payment business models. *Information Systems and E-Business Management*, *7*(3), 363–393. https://doi.org/10.1007/s10257-008-0098-9

Puschmann, T. (2017). Fintech. *Business & Information Systems Engineering*, *59*(1), 69–76. https://doi.org/10.1007/s12599-017-0464-6

Ralcheva, A., & Roosenboom, P. (2020). Forecasting success in equity crowdfunding. *Small Business Economics*, *55*(1), 39–56. https://doi.org/10.1007/s11187-019-00144-x

Richins, G., Stapleton, A., Stratopoulos, T. C., & Wong, C. (2017). Big Data Analytics: Opportunity or Threat for the Accounting Profession? *Journal of Information Systems*, *31*(3), 63–79. https://doi.org/10.2308/isys-51805

Rieger, P., Gewald, H., & Schumacher, B. (2013). Cloud-Computing in Banking: Influential Factors, Benefits and Risks from a Decision Maker's Perspective. In *Proceedings of the 19th Americas Conference on Information Systems,* Chicago, IL, USA.

Rimba, P., Tran, A. B., Weber, I., Staples, M., Ponomarev, A., & Xu, X. (2020). Quantifying the Cost of Distrust: Comparing Blockchain and Cloud Services for Business Process Execution. *Information Systems Frontiers*, *22*(2), 489–507. https://doi.org/10.1007/s10796-018-9876-1

Roma, P., Messeni Petruzzelli, A., & Perrone, G. (2017). From the crowd to the market: The role of reward-based crowdfunding performance in attracting professional investors. *Research Policy*, *46*(9), 1606–1628. https://doi.org/10.1016/j.respol.2017.07.012

Rose, S., Wentzel, D., Hopp, C., & Kaminski, J. (2021). Launching for success: The effects of psychological distance and mental simulation on funding decisions and crowdfunding performance. *Journal of Business Venturing*, *36*(6), 106021. https://doi.org/10.1016/j.jbusvent.2020.106021

Rukanova, B., Reuver, M. de, Henningsson, S., Nikayin, F., & Tan, Y.‑H. (2020). Emergence of collective digital innovations through the process of control point driven network reconfiguration and reframing: the case of mobile payment. *Electronic Markets*, *30*(1), 107–129. https://doi.org/10.1007/s12525-019-00352-z

Ryoba, M. J., Qu, S., & Zhou, Y. (2021). Feature subset selection for predicting the success of crowdfunding project campaigns. *Electronic Markets*, *31*(3), 671–684. https://doi.org/10.1007/s12525-020-00398-4

Schückes, M., & Gutmann, T. (2021). Why do startups pursue initial coin offerings (ICOs)? The role of economic drivers and social identity on funding choice. *Small Business Economics*, *57*(2), 1027–1052. https://doi.org/10.1007/s11187-020-00337-9

Scott, S. V., van Reenen, J., & Zachariadis, M. (2017). The long-term effect of digital innovation on bank performance: An empirical study of SWIFT adoption in financial services. *Research Policy*, *46*(5), 984–1004. https://doi.org/10.1016/j.respol.2017.03.010

Serrano-Cinca, C., & Gutiérrez-Nieto, B. (2016). The use of profit scoring as an alternative to credit scoring systems in peer-to-peer (P2P) lending. *Decision Support Systems*, *89*, 113–122. https://doi.org/10.1016/j.dss.2016.06.014

Shafi, K. (2021). Investors’ evaluation criteria in equity crowdfunding. *Small Business Economics*, *56*(1), 3–37. https://doi.org/10.1007/s11187-019-00227-9

Shao, Z., Zhang, L., Li, X [Xiaotong], & Guo, Y. (2019). Antecedents of trust and continuance intention in mobile payment platforms: The moderating effect of gender. *Electronic Commerce Research and Applications*, *33*, 100823. https://doi.org/10.1016/j.elerap.2018.100823

Shin, D.‑H. (2010). Modeling the Interaction of Users and Mobile Payment System: Conceptual Framework. *International Journal of Human-Computer Interaction*, *26*(10), 917–940. https://doi.org/10.1080/10447318.2010.502098

Singh, N., & Sinha, N. (2020). How perceived trust mediates merchant's intention to use a mobile wallet technology. *Journal of Retailing and Consumer Services*, *52*, 101894. https://doi.org/10.1016/j.jretconser.2019.101894

Skirnevskiy, V., Bendig, D., & Brettel, M. (2017). The Influence of Internal Social Capital on Serial Creators’ Success in Crowdfunding. *Entrepreneurship Theory and Practice*, *41*(2), 209–236. https://doi.org/10.1111/etap.12272

Stanko, M. A., & Henard, D. H. (2017). Toward a better understanding of crowdfunding, openness and the consequences for innovation. *Research Policy*, *46*(4), 784–798. https://doi.org/10.1016/j.respol.2017.02.003

Staykova, K., & Damsgaard, J. (2014). A Model of Digital Payment Infrastructure Formation and Development: The EU Regulator´s Perspective. In *Proceedings of the 2014 International Conference on Mobile Business,* London, United Kingdom.

Staykova, K. S., & Damsgaard, J. (2015). Introducing Reach And Range For Digital Payment Platforms. In *Proceedings of the 2015 International Conference on Mobile Business,* Forth Worth, TX, USA.

Steigenberger, N. (2017). Why supporters contribute to reward-based crowdfunding. *International Journal of Entrepreneurial Behavior & Research*, *23*(2), 336–353. https://doi.org/10.1108/IJEBR-04-2016-0117

Tao, Q., Dong, Y., & Lin, Z [Ziming] (2017). Who can get money? Evidence from the Chinese peer-to-peer lending platform. *Information Systems Frontiers*, *19*(3), 425–441. https://doi.org/10.1007/s10796-017-9751-5

Tauchert, C., & Mesbah, N. (2019). Following the Robot? Investigating Users’ Utilization of Advice from Robo-Advisors. In *Proceedings of the 40th International Conference on Information Systems,* Munich, Germany.

Thitimajshima, W., Esichaikul, V., & Krairit, D. (2018). A framework to identify factors affecting the performance of third-party B2B e-marketplaces: A seller’s perspective. *Electronic Markets*, *28*(2), 129–147. https://doi.org/10.1007/s12525-017-0256-3

Treiblmaier, H., Pinterits, A., & Floh, A. (2008). Success factors of internet payment systems. *International Journal of Electronic Business*, *6*(4), Article 20675, 369. https://doi.org/10.1504/IJEB.2008.020675

Trenz, M., Huntgeburth, J., & Veit, D. (2013). The Role of Uncertainty in Cloud Computing Continuance: Antecedents, Mitigators, and Consequences. In *Proceedings of the 21st European Conference on Information Systems,* Utrecht, The Netherlands.

Trütsch, T. (2016). The impact of mobile payment on payment choice. *Financial Markets and Portfolio Management*, *30*(3), 299–336. https://doi.org/10.1007/s11408-016-0272-x

Underwood, S. (2016). Blockchain beyond bitcoin. *Communications of the ACM*, *59*(11), 15–17. https://doi.org/10.1145/2994581

van Alstyne, M. (2014). Why Bitcoin has value. *Communications of the ACM*, *57*(5), 30–32. https://doi.org/10.1145/2594288

Vismara, S. (2016). Equity retention and social network theory in equity crowdfunding. *Small Business Economics*, *46*(4), 579–590. https://doi.org/10.1007/s11187-016-9710-4

Vismara, S. (2018). Information Cascades among Investors in Equity Crowdfunding. *Entrepreneurship Theory and Practice*, *42*(3), 467–497. https://doi.org/10.1111/etap.12261

WAKAMORI, N., & WELTE, A. (2017). Why Do Shoppers Use Cash? Evidence from Shopping Diary Data. *Journal of Money, Credit and Banking*, *49*(1), 115–169. https://doi.org/10.1111/jmcb.12379

Walther, S., Sedera, D., Urbach, N., Eyman, T., Otto, B., & Sarker, S. (2018). Should We Stay or Should We Go? Analyzing Continuance of Cloud Enterprise Systems. *Journal of Information Technology Theory and Application*, *19*(2), 57–88.

Wang, N [Nianxin], Li, Q., Liang, H., Ye, T., & Ge, S. (2018). Understanding the importance of interaction between creators and backers in crowdfunding success. *Electronic Commerce Research and Applications*, *27*, 106–117. https://doi.org/10.1016/j.elerap.2017.12.004

Wang, Z., Jiang, C., Ding, Y., Lyu, X., & Liu, Y [Yao] (2018). A Novel behavioral scoring model for estimating probability of default over time in peer-to-peer lending. *Electronic Commerce Research and Applications*, *27*, 74–82. https://doi.org/10.1016/j.elerap.2017.12.006

Watson, H. J. (2018). Revisiting Ralph Sprague’s Framework for Developing Decision Support Systems. *Communications of the Association for Information Systems*, *42*, 363–385. https://doi.org/10.17705/1CAIS.04213

Weaver, N. (2018). Risks of cryptocurrencies. *Communications of the ACM*, *61*(6), 20–24. https://doi.org/10.1145/3208095

Wessel, M., Thies, F., & Benlian, A. (2016). The emergence and effects of fake social information: Evidence from crowdfunding. *Decision Support Systems*, *90*, 75–85. https://doi.org/10.1016/j.dss.2016.06.021

Wessel, M., Thies, F., & Benlian, A. (2017). Opening the Floodgates: The Implications of Increasing Platform Openness in Crowdfunding. *Journal of Information Technology*, *32*(4), 344–360. https://doi.org/10.1057/s41265-017-0040-z

Wiener, M., Saunders, C., & Marabelli, M. (2020). Big-data business models: A critical literature review and multiperspective research framework. *Journal of Information Technology*, *35*(1), 66–91. https://doi.org/10.1177/0268396219896811

Wooder, S., & Baker, S. (2012). Extracting Key Lessons in Service Innovation. *Journal of Product Innovation Management*, *29*(1), 13–20. https://doi.org/10.1111/j.1540-5885.2011.00875.x

Xie, K., Liu, Z., Chen, L., Zhang, W., Liu, S., & Chaudhry, S. S. (2019). Success factors and complex dynamics of crowdfunding: An empirical research on Taobao platform in China. *Electronic Markets*, *29*(2), 187–199. https://doi.org/10.1007/s12525-018-0305-6

Xu, Q., & Riedl, R. (2011). Understanding Online Payment Method Choice: An Eye-tracking Study. In *Proceedings of the 32nd International Conference on Information Systems,* Shanghai, China.

Ye, X., Dong, L., & Da Ma (2018). Loan evaluation in P2P lending based on Random Forest optimized by genetic algorithm with profit score. *Electronic Commerce Research and Applications*, *32*, 23–36. https://doi.org/10.1016/j.elerap.2018.10.004

Yu, S., Johnson, S [Scott], Lai, C., Cricelli, A., & Fleming, L. (2017). Crowdfunding and regional entrepreneurial investment: an application of the CrowdBerkeley database. *Research Policy*, *46*(10), 1723–1737. https://doi.org/10.1016/j.respol.2017.07.008

Yuan, H., Lau, R. Y., & Xu, W. (2016). The determinants of crowdfunding success: A semantic text analytics approach. *Decision Support Systems*, *91*, 67–76. https://doi.org/10.1016/j.dss.2016.08.001

Yuan, Y., Lai, F., & Chu, Z. (2019). Continuous usage intention of Internet banking: a commitment-trust model. *Information Systems and E-Business Management*, *17*(1), 1–25. https://doi.org/10.1007/s10257-018-0372-4

Zarmpou, T., Saprikis, V., Markos, A., & Vlachopoulou, M. (2012). Modeling users’ acceptance of mobile services. *Electronic Commerce Research*, *12*(2), 225–248. https://doi.org/10.1007/s10660-012-9092-x

Zhang, J. M., Zhu, S. Z., Yan, W., & Li, Z. P. (2020). RETRACTED ARTICLE: The construction and simulation of internet financial product diffusion model based on complex network and consumer decision-making mechanism. *Information Systems and E-Business Management*, *18*(4), 545–555. https://doi.org/10.1007/s10257-018-0384-0

Zheng, H., Li, D., Wu, J., & Xu, Y. (2014). The role of multidimensional social capital in crowdfunding: A comparative study in China and US. *Information & Management*, *51*(4), 488–496. https://doi.org/10.1016/j.im.2014.03.003

Zheng, H., Xu, B., Wang, T [Tao], & Chen, D [Dongyu] (2017). Project Implementation Success in Reward-Based Crowdfunding: An Empirical Study. *International Journal of Electronic Commerce*, *21*(3), 424–448. https://doi.org/10.1080/10864415.2016.1319233

Zhou, B., & Buyya, R. (2019). Augmentation Techniques for Mobile Cloud Computing. *ACM Computing Surveys*, *51*(1), 1–38. https://doi.org/10.1145/3152397

Zhou, M. J., Lu, B., Fan, W. P., & Wang, G. A. (2018). Project description and crowdfunding success: An exploratory study. *Information Systems Frontiers*, *20*(2), 259–274. https://doi.org/10.1007/s10796-016-9723-1

Zhou, T. (2013). An empirical examination of continuance intention of mobile payment services. *Decision Support Systems*, *54*(2), 1085–1091. https://doi.org/10.1016/j.dss.2012.10.034

Zohar, A. (2015). Bitcoin: Under the Hood. *Communications of the ACM*, *58*(9), 104–113. https://doi.org/10.1145/2701411

1. This article was retracted by the journal where it was published. This happened after acceptance of our study. Therefore, it is still included in our analysis. [↑](#footnote-ref-2)
